# Examination of the temporal and spatial dynamics of the gut microbiome in newborn piglets reveals distinct microbial communities in six intestinal segments

**DOI:** 10.1038/s41598-019-40235-z

**Published:** 2019-03-05

**Authors:** Ying Liu, Zhijun Zheng, Lihuai Yu, Sen Wu, Li Sun, Shenglong Wu, Qian Xu, Shunfeng Cai, Nan Qin, Wenbin Bao

**Affiliations:** 1 College of Animal Science and Technology, Yangzhou University, Yangzhou, 225009 China; 2 School of Life Science, Huaiyin Normal University, Huaian, 223001 China; 3 Realbio Genomics Institute, Shanghai, 200123 China; 4 Shenzhen Jinrui Biotechnology, Co. Ltd., Shenzhen, 518000 China; 5 Joint International Research Laboratory of Agriculture & Agri-Product Safety, Yangzhou University, Yangzhou, 225009 China

## Abstract

Intestinal microbiota plays a crucial role in immune development and disease progression in mammals from birth onwards. The gastrointestinal tract of newborn mammals is rapidly colonized by microbes with tremendous biomass and diversity. Understanding how this complex of segmental communities evolves in different gastrointestinal sites over time has great biological significance and medical implications. However, most previous reports examining intestinal microbiota have focused on fecal samples, a strategy that overlooks the spatial microbial dynamics in different intestinal segments. Using intestinal digesta from six intestinal segments (duodenum, jejunum, ileum, cecum, colon and rectum) of newborn piglets, we herein conducted a large-scale 16S rRNA gene sequencing-based study to characterize the segmental dynamics of porcine gut microbiota at eight postnatal intervals (days 1, 7, 14, 21, 28, 35, 120 and 180). A total of 4,465 OTUs were obtained and showed that the six intestinal segments could be divided into three parts; in the duodenum-jejunum section, the most abundant genera included *Lactobacillus* and *Bacteroides*; in the ileum, *Fusobacterium* and *Escherichia*; and in the cecum-rectum section, *Prevotella*. Although the microbial communities of the piglets were similar among the six intestinal segments on postnatal day 1, they evolved and quickly differentiated at later intervals. An examination of time-dependent alterations in the dominant microbes revealed that the microbiome in the large intestine was very different from and much more stable than that in the small intestine. The gut microbiota in newborn piglets exhibited apparent temporal and spatial variations in different intestinal segments. The database of gut microbes in piglets could be a referable resource for future studies on mammalian gut microbiome development in early host growth phases.

## Introduction

Over the last decade, influence of gut microbiota on host health has become increasingly recognized, as a growing body of evidence has revealed that intestinal microbiota plays important roles in immunity development, vitamin synthesis, digestion and the modulation of host gene expression^1–4^. Many chronic human diseases, such as obesity^5^, diabetes^6,7^, cirrhosis^8^, rheumatoid arthritis^9^ and inflammatory bowel disease^10^, are associated with alterations in gut microbial communities. Although chronic diseases typically affect adults, their pathogenic roots may start much earlier, which is implied by their connections with the gut microbiome. Infants are born germfree or with little microbial presence^11^, but microbes quickly colonize many sites, and the gut can have profound and lasting effects on the host^12^. Thus far, it has not been fully understood how various microbes establish populations in different intestinal segments of newborn infants, as analyses using fecal samples are currently the approach for microbiome-based research^13–17^, are inadequate for elucidating the spatial dynamics of gut microbiota. In addition, some intestinal segments are also poorly accessible through gastroduodenoscopy and colonoscopy practices^18–20^. Therefore, the microbial colonization of different intestinal segments during early growth stages remain poorly understood in humans. A practical approach to investigate this question is to use an animal model, and the pig is an excellent model for studying the human gut microbiome due to human-pig similarities in gut metagenomes^21^, gut microbial compositions^22^, and many disease-associated alleles^23^.

Here, we investigated the dynamic landscape of the porcine gut microbiota in six intestinal segments (duodenum, jejunum, ileum, cecum, colon and rectum) at eight postnatal intervals (postnatal day 1, 7, 14, 21, 28, 35, 120 and 180), in which we examined the segmental bacterial composition and abundance in the gut. Our analyses generated a comprehensive database of gut microbes in piglets at early growth phases. Importantly, our data revealed time-dependent differences in microbial communities among individual intestinal segments in newborn mammals.

## Materials and Methods

### Ethics statement

The Institutional Animal Care and Use Committee (IACUC) of Yangzhou University Animal Experiments Ethics Committee approved the animal study proposal with the permit number: SYXK(Su) IACUC 2012-0029. All animal experimental procedures were performed in accordance with the Regulations for the Administration of Affairs Concerning Experimental Animals approved by the State Council of the People’s Republic of China.

### Animal collection and sample collection

All Meishan piglets were collected from Kunshan Conservation Ltd. (Suzhou City, Jiangsu Province, China). Meishan is a well-established local Chinese breed known for its high fecundity, strong immune response and high meat quality. For this study, we chose five multiparous Meishan sows that were similar in age, weight, and body shape and had farrowed on the same day. The piglets received the same diet and were housed in an environmentally controlled room. After parturition, a total of 78 newborn piglets were obtained, with an average of 15 or more piglets per litter. We designated the first day of the newborns as day 1. Once the sows farrowed, one piglet from each litter was immediately chosen to be slaughtered within an hour. A total of five piglets (three females and two males) of similar weights were sacrificed. Following slaughter, samples of the digesta from the middle of the duodenum, jejunum, ileum, cecum, colon and rectum were collected simultaneously and snap-frozen in liquid nitrogen. The remaining piglets were housed in five pens in an environmentally controlled room and were fed with a standard swine diet under identical husbandry conditions until weaning (day 35). After weaning, 3 piglets of the same litter were housed in one pen. Similar to the aforementioned sample collection procedure on day 1, one piglet from each litter was chosen to be sacrificed at postnatal days 7, 14, 21, 28, 35, 120 and 180 after farrowing using an intravenous injection of pentobarbital sodium, which minimized animal suffering. The piglets chosen at each interval were matched in weight and body shape. At each time point, the ratio of male to female piglets was 2:3 or 3:2, and the final gender ratio was 1:1. Following slaughter, samples of the digesta from the middle of the duodenum, jejunum, ileum, cecum, colon and rectum were collected from each animal simultaneously. A total of 240 samples were collected (five individuals at each of eight development stages; and for each individual, digesta was collected from six intestinal segments) and snap-frozen in liquid nitrogen. All the piglets were weaned on postnatal day 35. After weaning, the pigs were fed with grower diets (feed formulation prepared in our laboratory). The ingredients of the diets are provided in Table S1.

### Amplification and sequencing of 16S rRNA gene fragments

Microbial genomic DNA was extracted from intestinal digesta samples using the QIAamp DNA Stool Mini Kit (Qiagen, Germany) according to the manufacturer’s instructions. DNA integrity and purity was assessed by 1% agarose gel electrophoresis and a NanoDrop 8000 spectrophotometer (Thermo Fisher Scientific, USA).

DNA sequencing libraries targeting the V3–V4 hypervariable region of 16S rRNA gene were prepared by PCR amplification using specific primers supplemented with Illumina sequencing adapters and sample-specific barcodes according to Illumina’s instructions (https://support.illumina.com/downloads/16s_metagenomic_sequencing_library_preparation.html). The primers used were 341F (5′-ACTCCTACGGGRSGCAGCAG) and 806R (5′-GGACTACVVGGGTATCTAATC)^24^. PCRs were performed using the KAPA HiFi Hotstart Readymix PCR kit (KAPA Biosystems, USA) according to Illumina’s instructions as mentioned above. After amplification, the libraries were purified using 2% agarose gel electrophoresis and the AxyPrep DNA gel extraction kit (Axygen, USA). The DNA concentration of each library was determined using the Qubit^®^ dsDNA HS Assay kit (Invitrogen). All libraries were pooled and sequenced using an Illumina HiSeq Rapid SBS Kit V2 on the Hiseq2500 platform with PE250 mode at Realbio Technology Co., Ltd. (Shanghai, China).

### Process of sequencing data

16S rRNA gene sequences were trimmed of barcodes and primers^25^. Reads with an overlap longer than 10 bp were merged. The resulting 16S rRNA gene sequences were restricted between 220 bp and 500 bp such that the average Phred score of bases was no worse than 20 (Q20) and there were no more than 3 ambiguous N. The copy number of tags was enumerated and redundancy of repeated tags was removed. Only the tags with frequency more than 1, which tend to be more reliable, were clustered into OTUs, each of which had a representative tag.

### Taxonomy classification and statistical analysis

After discarding singleton sequences, the high-quality reads were clustered into operation taxonomic units (OTUs) using Usearch (v7.0.1090) in QIIME (v1.9.1, http://qiime.org/scripts/pick_otus.html)^26^ with a similarity threshold of 0.97. Taxonomy was assigned to the OTUs using the RDP classifier. All the samples were randomly subsampled to an even depth (27,607 reads) prior to the calculation of alpha and beta diversity metrics, and the number of sequences in each sample are shown in Table S2. Alpha diversity was assessed by Shannon diversity index, and beta diversity by weighted UniFrax distances and principal coordinate analysis (PCoA). Both alpha diversity and beta diversity were calculated in QIIME. Weighted UniFrac distances were calculated using R (v.3.1.0) with the library vegan (version number) and an ANOSIM performed on these distances by intestinal section and age.

## Results

### Gut microbiota is distributed differentially among individual intestinal segments

**Figure 1:**
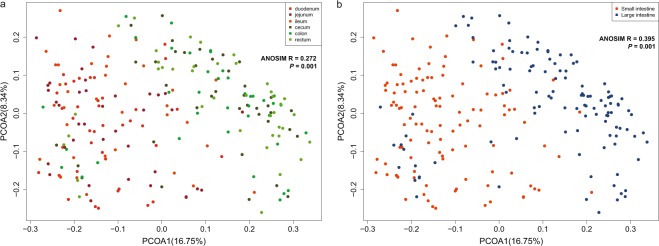
Principal coordinate analysis (PCoA) of all 230 samples based on weighted UniFrac distances among different intestinal segments. (**a**) The samples in different intestinal segments are colored differentially (duodenum: red, ileum: orange, jejunum: dark read, cecum: dark green, colon: green; rectum: light green). (**b**) The samples in the small intestine (duodenum, ileum and jejunum) were colored in red, and those in the large intestine (cecum, colon and rectum) are colored in blue.

A landscape of porcine gut microbiota in six intestinal segments (duodenum, jejunum, ileum, cecum, colon and rectum) was revealed by profiling of the taxonomic abundance in all samples (Table S3). Principal coordinates analysis (PCoA) showed the structural differences in the microbiota based on intestinal segment (Fig. 1). The Weighted UniFrac distances (ANOSIM R = 0.272, *p* = 0.001, Fig. S1) and Bray-Curtis dissimilarities (Fig. S2) showed that gut microbiota distributed differentially in six intestinal segments (Fig. 1a). Overall, the six segments could be divided into two distinct groups, with the duodenum, jejunum and ileum comprising one, and the cecum, colon and rectum comprising the other; the two groups displayed apparent differences (ANOSIM R = 0.395, *p* = 0.001, Figs 1b and S3). This pattern is clearly consistent with gut anatomy in that the duodenum, jejunum and ileum from the small intestine, and the cecum, colon and rectum from the large intestine. The Bray-Curtis dissimilarities (Fig. S4) also showed distinct differences in the gut microbiome between the small intestine and the large intestine. The differentiation between the small intestine and the large intestine could also be illustrated by the gut microbial compositions (Fig. S5). For example, the most abundant genera in the large intestine were *Prevotella* (days 7, 21, 28, 35, 120 and 180 in the cecum, colon and rectum), *Bacteroides* (day 1 in the cecum, colon and rectum) and *Fusobacterium* (day 14 in the cecum, colon and rectum), whereas those in the small intestine were *Lactobacillus* (days 21, 35, and 120 in the duodenum; days 7 and 120 in the jejunum), *Escherichia* (day 1 in the duodenum, jejunum and ileum; days 7, 21, 28, 35, and 120 in the ileum). We also noticed that in the small intestine, there were apparent microbial differences between duodenum and jejunum in the one part and ileum in the other, as *Lactobacillus* was dominant in the duodenum and jejunum but not the ileum, whereas a significant level of *Fusobacterium* was present only in the ileum. Lastly, our data also showed that over the course of this study, the composition of the large intestinal microbiome was considerably more stable than that of the small intestinal microbiome (Fig. S5).

### The dynamic landscape of porcine gut microbiota in six intestinal segments at eight postnatal intervals

We next analyzed the time-dependent dynamics of intestinal microbial communities. The Weighted UniFrac distances (ANOSIM R = 0.18, *p* = 0.001, Fig. S6) and Bray-Curtis dissimilarities (Fig. S7) showed that gut microbiota were quite different at the eight different postnatal intervals. A landscape of porcine gut microbiota in eight growth stages (days 1, 7, 14, 21, 28, 35, 120 and 180) was shown by the profiling of the OTU abundance in all the samples (Table S4). The overall temporal dynamics of the porcine gut microbiota are shown in Fig. S5. The gut microbiota varied considerably with time in different intestinal segments, especially between the small intestine and large intestine. In the small intestine, the diversity of the gut microbiome, represented by the Shannon diversity index, decreased from postnatal day 1 to day 14 but increased from postnatal day 14 to days 35 and 120 (Fig. S8a). In the large intestine, by comparison, the diversity of the gut microbiome increased from day 1 to day 35 and increased from day 35 to day 180 (Fig. S8b); the results showed that the diversity of the gut microbiome in the large intestine increased during the early postnatal period. Interestingly, the Shannon diversity index was higher in the small intestine than in the large intestine at most time points; although at later stages, this parameter was greater in the large intestine (Fig. S8). In summary, the changes to the gut microbiome in the large intestine were different from those in the small intestine.

**Figure 2:**
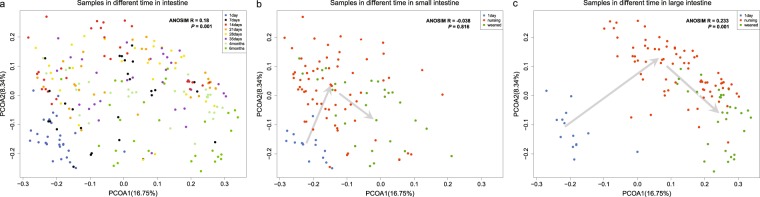
Weighted UniFrac PCoA of different growth stages. (**a**) PCoA of all 230 samples according to different growth stages; the points with different colors represent the different samples from postnatal days 1, 7, 14, 21, 28, 35, 120 and 180. (**b,c**) PCoA of the 113 samples in the small intestine (**b**) and the 117 samples in the large intestine (**c**). The colors represent the different time points; 1 day: blue, nursing (postnatal days 7, 14, 21, 28, and 35): red, weaning (postnatal day 120 and day 180): green, Each ‘arrow’, generated according to the centroid of the sampling points at each interval, illustrates the changing trend over time.

The results revealed that tremendous changes in the gut microbiome occurred at the eight postnatal intervals (ANOSIM R = 0.18, *p* = 0.001, Figs 2 and S6). The samples from the gut microbiome on postnatal day 1 showed clear clustering (Fig. 2a), whereas the samples on later postnatal days displayed a relatively wide spatial distribution (Fig. 2a), which is indicative of the differential microbiome compositions in individual intestinal segments after day 1 (Fig. 2b,c). In addition, during the nursing (postnatal day 7 to day 35) and weaning periods (postnatal day 120 to day 180), the evolution of microbial communities in the small intestine was considerably different from the corresponding events in the large intestine. In the small intestine, the changes in microbial communities from day 1 to the nursing period and weaning period were relatively small (Fig. 2b). The Weighted UniFrac distances (ANOSIM R = −0.038, *p* = 0.816, Fig. S9) and the Bray-Curtis dissimilarities (Fig. S10) also showed that gut microbiota varied little among day 1, nursing period and weaning period in the small intestine. However, in the large intestine, the changes in microbial communities from day 1 to the nursing period and weaning period were relatively large (Fig. 2c). The Weighted Unifrac distances (ANOSIM R = 0.233, *p* = 0.001, Fig. S11) and the Bray-Curtis dissimilarities (Fig. S12) showed that the gut microbiota were obviously different among day 1, nursing period and weaning period in the large intestine. Overall, our data showed that the microbial communities in individual intestinal segments were highly dynamic.

### Variations in the dominant microbiota over time in the small and large intestines during early growth stages

We next examined microbial differences at the genus level between the small and large intestines (Fig. S13). We combined all data from the duodenum, jejunum and ileum to represent the small intestine and data from the cecum, colon and rectum to represent the large intestine. On postnatal day 1, the dominant microbes were similar between the small intestine and large intestine, as *Escherichia*/*Shigella, Bacteroides* and *Veillonella* were the dominant bacteria in both the small and large intestines. On postnatal day 7, the microbial compositions changed tremendously from those on postnatal day 1, resulting in a clear difference in microbiome composition between the small and large intestines. Specifically, on postnatal day 7, *Lactobacillus*, *Escherichia*/*Shigella*, and *Bacteroides* were the most abundant taxa in the small intestine, whereas *Prevotella* was dominant and accounted for almost half of the microbial abundance in the large intestine. On postnatal day 14, *Fusobacterium* displayed a clear increase in abundance in both the small and large intestines. After postnatal day 21, *Prevotella* consistently remained as the dominant microbe in the large intestine, whereas *Lactobacillus*, *Prevotella* or *Escherichia*/*Shigella* were the most abundant species in the small intestine.

A comparison of the data between postnatal day 1 and day 180 revealed that in the small intestine, the abundance of *Prevotella* and *Fusobacterium* on postnatal day 1 was apparently lower than that 6 months later. Conversely, *Bacteroides* accounted for 9.86% of the abundance on postnatal day 1, but the proportion decreased to only 4.10% 6 months later. In the large intestine, the relative abundance of *Bacteroide*s decreased from 27.82% to 4.52%, as did that of *Clostridium sensu stricto* from 10.5% to almost 0%. As a consequence, the dominant status of *Bacteroide*s and *Clostridium sensu stricto* on postnatal day 1 was replaced by *Prevotella* (12.47%) and *Alloprevotella* (5.13%) 6 months later (Fig. S13).

### Variations in bacteria before and after weaning in intestinal segments

Mammals experience their first change in nutrition intake when they are born, at which point they switch from placenta feeding to breastfeeding. Our results therefore indicated that the postpartum dietary transition had a great impact on intestinal microbial composition, as the microbial compositions were similar on postnatal day 1 but differed considerably from day 7 onwards (Figs 2 and S5). Furthermore, when newborn piglets mature, they transition from breastfeeding to consuming solids. These diet changes may correlate with extraordinary alterations in the gut microbiome among individual intestinal segments. To examine this diet transition-associated microbiome change, all the test animals were subject to the following feeding arrangement: the piglets were not fed until one hour after birth, at which point they were breastfed until day 35 and were subsequently weaned and received fodder feeding for the remaining period of the study.

**Figure 3:**
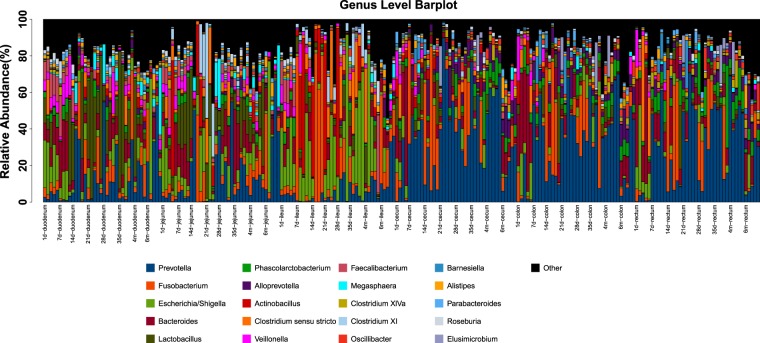
Bar plot of the microbial compositions of the top 20 genera among 230 samples. The horizontal axis represents the different samples; the vertical axis represents the relative abundance of the different genera. Different colors represent different bacterial genera.

*Lactobacillus* has been reported to be closely associated with milk digestion^27^. In this study, our data revealed that the relative abundance of *Lactobacillus* was highest in the duodenum and jejunum (Fig. 3) and that its abundance remained relatively high during the breastfeeding period (7 days, 14 days, 21 days and 28 days) but drastically decreased during the weaning period (postnatal day 35 and onward) (Fig. S14a). Both *Bacteroides* and *Prevotella* are well known fermenters of dietary fiber^28^. In this study, *Bacteroides* was the most abundant in the intestinal segments on postnatal day 1 and day 7 (Fig. 3), but afterwards, this dominant position was replaced by *Prevotella* (Fig. S14b). *Veillonella* is a commensal species in the oral, gastrointestinal, respiratory, and genitourinary tracts in humans and animals^29^. Interestingly, our results showed that the relative abundance curve of *Veillonella* was opposite of that of *Lactobacillus* in the duodenum and cecum (Fig. S15a,b). The results suggested that *Veillonella* was also associated with the aforementioned diet transition in piglets.

In addition to the microbial shifts that occurred at the two points of nutrition intake changes, our data also showed that some microbes displayed dramatic abundance alterations at other time points. Before weaning, piglets acquire nutrition through breastfeeding. Interestingly, we found that the significant increase of *Fusobacterium* and *Clostridium* occurred on postnatal day 14 (Fig. S16), which was in the middle of the breastfeeding period.

## Discussion

The gut microbiota plays an important role in human health, and microbiota aberrations are associated with many chronic diseases^30^. In this study, we used newborn piglets as the experimental model to study the temporal dynamics of microbiota in six intestinal segments. Our data revealed extraordinary temporal and spatial dynamics of intestinal microbial communities in newborn mammals.

Our results showed that the microbes were present in all intestinal segments on day 1. This finding appears to contradict the conventional perception regarding the prenatal gut microbiota in mammals. It is commonly believed that the intestine of mammalian fetus is sterile^31^. After birth, the newborn is quickly colonized by a variety of microorganisms by interacting with the environment^32–34^. However, recent studies have shown that some microbes can be detected in fetal meconium^35^, which is in agreement with our data on day 1. It was proposed that most of these colonized microorganisms originate from the mother’s vagina, uterus, and mouth^36–39^_._ As shown in Fig. S17, upon birth, the dominant phyla in Meishan pigs are *Bacteroidetes*, *Firmicutes*, *Proteobacteria*, *Fusobacteria*, and *Actinobacteria*, which are very similar to those of newborn infants^22^. After reaching adulthood, the gut microbial composition is very similar to those of other pig breeds, such as Jinhua and Landrance pigs^40^.

A previous study on intestinal microbiota in pigs showed that the small and large intestines had apparent differences in microbial compositions^15^. Such patterns were also found in this study. However, our results also showed that the small intestine could be divided into two microbiota zones, the duodenum and jejunum (in which *Lactobacillus* and *Bacteroides* were more abundant) and the ileum (in which there was a significant presence of *Fusobacterium* and *Escherichia*) (Figs 3 and S4). We speculate that the ileum, located between the small intestine and large intestine, has some unique anatomical features to accommodate a distinct microbial composition.

Although most digestive function is conferred by digestive endogenous enzymes produced by the host^41–43^, the gut microbiota also plays an indispensable role^44,45^. Looft *et al*.^46^ found that the microbial communities in the large intestine harbor a large pool of genes for breaking down cell wall components and that these genes were lacking in the ileum. This finding also implies that in the digestive tract of mammals, the small intestine and large intestine, along with the microbes inhabiting the two subdomains, play very different roles in digestion and nutrient absorption. This study generated some interesting findings from the gut microbiota of neonatal mammals, such as those contributing to digestion, and suggest a process as below: After the initial digestion of food in the stomach, the resulting chyme enters the small intestine, where it is subjected to further breakdown that requires a myriad of microbes, likely including *Lactobacillus*, *Bacteroides*, and *Fusobacterium*. These bacteria are involved in degrading carbohydrates, proteins, and fat into small molecules that can be easily absorbed through the epithelium of the small intestine. In addition, the dietary fibers, proteins and peptides that escape digestion in the small intestine are metabolized by some microbes in the lower gut. In the colon, the local microbes can metabolize otherwise non-digestible content into short-chain fatty acids and other small-molecule nutrients. For example, *Prevotella* is important for the digestion of those dietary fibers and is dominant in the large intestine. However, the large intestine has limited digestive capability^47^, where only approximately 10% of nutrient absorption occurs, mostly in the colon^48,49^.

The functional differences between the small and large intestines suggest significant microbiome composition divergence, which were confirmed by the PCoA and composition differences identified in this study (Figs 1 and 3). In duodenum and jejunum, the dominant genera were *Lactobacillus* and *Bacteroides* (Fig. 3)*. Lactobacillus* species constitute a major phylotypic group in the proximal region of gastrointestinal tract in several mammals as well as in chicken^50–52^ and metabolize carbohydrates to produce lactic acid as a major end-product^53,54^. *Lactobacillus* abundance is associated with milk intake^27^. *Bacteroides* has been reported to be associated with a high protein diet in humans^55,56^. In the ileum, our data revealed that *Fusobacterium* and *Escherichia/Shigella* were the dominant genera (Fig. S5); this finding was consistent with previous findings that they both belonged to the normal human microbiota^57,58^ and suckling Landrace piglets^59^. Under normal circumstances, many species in the two genera are commensals, and some strains of *Fusobacterium* can produce butyrate from carbohydrates^60^. Certain *Escherichia* strains have developed a symbiotic relationship with anaerobes that require mono- and disaccharides as well as anaerobes that degrade complex polysaccharides^61^. In addition, *Escherichia spp*. are involved in scavenging oxygen to contribute to an anaerobic environment^62,63^. These activities may explain the dominant level of *Escherichia* in the infant gut^64,65^. Nevertheless, some strains of *Fusobacterium* and *Escherichia* are pathogenic and are associated with severe gastrointestinal diseases including inflammatory bowel disease^66^ and diarrhea^67^, providing a new insight into the interplay between gut microbiota components and host immune system^68^. In the large intestine, *Prevotella* remained dominant at most time points (Fig. 3). *Prevotella* can degrade the dietary fiber of the plant cell wall to produce short chain fatty acids^69^. A *Prevotella*-dominated microbiota was reported to be associated with a high-fiber diet in humans^55,56^, which is in agreement with its high abundance during the weaning period of the piglets when they received fodder feed (Fig. S13).

Our examination of the temporal dynamics of the microbial communities in the intestinal segments showed that the means of nutrition intake had a great impact on the development of gut microbiota. For example, distinct microbial communities did not appear in individual intestinal segments on postnatal day 1 when the newborn host transitioned from placenta-feeding to breastfeeding, but they rapidly diverged at later. Moreover, weaning correlated with apparent changes in microbiota structure (Fig. 2b,c) and apparent abundance changes of the microbes associated with milk digestion (e.g., *Lactobacillus*) or fibers (e.g., *Bacteroides*, *Prevotella*). Hence, our findings corroborated a recent study^70^, which showed that the microbial communities in individual intestinal segments are closely intertwined, and possibly coevolved, with the host digestive functions within the intestinal tract.

Furthermore, we also showed that several genera contain potentially pathogenic species such as *Fusobacterium* and *Clostridium* underwent dramatic changes in relative abundance before weaning (Fig. S16a,b), which might be explained by their interactions with host immunity^71,72^. *Clostridia* is a major class of gut microbes that can interact with intestinal regulatory T (Treg) cells and correspondingly modulate inflammatory and allergic activities. However, the underlying mechanisms by which commensal microbes induce colonic Treg cells remain unclear^73^. The extraordinary abundance fluctuations in *Fusobacterium* and *Clostridium* reflect the complex host-microbial interactions that presumably contribute to immunity development in the gut.

In summary, our analyses revealed that the gut microbial communities in newborn piglets are highly dynamic in individual intestinal segments and are responsive to changes in nutrition intake. This work illustrated the tremendous volatility in the temporal and spatial patterns of gut microbiota in newborn mammals. Our findings demonstrated that analyses based on fecal samples are insufficient to investigate the complex intestinal microbial communities.

## Conclusions

Using newborn piglets, we herein showed that the six intestinal segments (duodenum, jejunum, ileum, cecum, colon and rectum) exhibited tremendous differences in microbial compositions and that these microbial compositions were highly dynamic during the eight different postnatal intervals. In addition, certain genera containing potentially pathogenic species were altered in relative abundance during piglet development. Our findings demonstrate that the intestinal microbiota is complex and can be influenced by other factors such as diet.

## Supplementary information

Supplementary info

## Data Availability

The sequencing data of the 230 samples have been submitted to the NCBI Sequence Read Archive under accession number SRP109947.
