# Supplementary material for: Examination of the temporal and spatial dynamics of the gut microbiome in newborn piglets reveals distinct microbial communities in six intestinal segments
Source: Sci Rep. 2019 Mar 5;9:3453. doi: 10.1038/s41598-019-40235-z (PMC6400902; doi:10.1038/s41598-019-40235-z)
Supplement: Supplementary file 1 — Supplementary info [file 41598_2019_40235_MOESM1_ESM.docx]

**Examination of the temporal and spatial dynamics of the gut microbiome in newborn piglets reveals distinct microbial communities in six intestinal segments**

Ying Liu^1,2†^, Zhijun Zheng^3,4†^, Lihuai Yu^1^, Sen Wu^1^, Li Sun^1^, Shenglong Wu^1,5^, Qian Xu^3,4^, Shunfeng Cai^3,4^, Nan Qin^3 ,4^ & Wenbin Bao^1,5^

^1^College of Animal Science and Technology, Yangzhou University, Yangzhou 225009, China. ^2^School of Life Science, Huaiyin Normal University, Huaian 223001, China. ^3^Realbio Genomics Institute, Shanghai, 200123, China. ^4^Shenzhen Jinrui Biotechnology, Co. Ltd., Shenzhen 518000, China. ^5^Joint International Research Laboratory of Agriculture & Agri-Product Safety, Yangzhou University, Yangzhou 225009, China.^†^Equal contributors

Ying Liu^1,2^

Email: [yddkly@163.com](mailto:yddkly@163.com)

Zhijun Zheng^3,4^

E-mail: zhengzj@realbio.cn

Lihuai Yu^1^

E-mail: lhyu@yzu.edu.cn

Sen Wu^1^

E-mail: 15705273995@163.com

Li Sun^1^

E-mail: sl19920327@163.com

Shenglong Wu^1,5^

E-mail: slwu@yzu.edu.cn

Qian Xu^3,4^

E-mail: xuq@realbio.cn

Shunfeng Cai^3,4^

E-mail: caisf@realbio.cn

Corresponding author: Wenbin Bao

Address: Key Laboratory for Animal Genetics, Breeding, Reproduction and Molecular Design of Jiangsu Province, College of Animal Science and Technology, Yangzhou University, Yangzhou, Jiangsu 225009, P. R. China;

Tel.: 86-514-87979350; Fax: 86-514-87350440; E-mail: wbbao@yzu.edu.cn

Nan Qin

Address: Realbio Genomics Institute, Shanghai, 200123, China; E-mail: qinnan001@126.com

**Supplementary information**

Table S1

Diet composition and nutrient levels

| Day 1 - Day 35 | Breastfeeding |  |
| --- | --- | --- |
| Day 36 - Day 180 | Grower diets (feed formulation prepared in our laboratory) | |
|  | Ingredient | Percentage (%) |
|  | Corn | 30 |
|  | Bran | 25 |
|  | Soybean meal | 14 |
|  | Green bran | 27 |
|  | Premix1) | 4 |
|  | Dietary nutrient levels2) | |
|  | Digestible energy | 12.77 MJ/kg |
|  | Crude protein | 15.53% |
|  | Crude fiber | 5.79% |
|  | Ca | 0.70% |
|  | P | 1.39% |
|  | Lysine | 0.71% |

1)The premix provided the following per kg of diets: Fe (as ferrous sulfate) 80 mg, Cu (as copper sulfate) 15 mg, Zn (as zinc sulfate) 80 mg, Mn (as manganese sulfate) 5 mg, Se (as sodium selenite) 0.10 mg, I (as potassium iodide) 0.10 mg, VA 4 480 IU, VD3 500 IU, VE 20 IU, VK3 2.20 mg, VB1 1.80 mg, VB2 2.20 mg, VB6 1.50 mg, VB12 12 ug, folic acid 0.30 mg, biotin 0.05 mg, nicotinic acid 10 mg, calcium pantothenate 8 mg.
2)Nutrient levels were calculated values.

Table S2

The numbers of Clean tags and data imformation in each sample.

| Sample name | Growth stages | Intestinal segments | Clean tags | Clean tags at each stage |
| --- | --- | --- | --- | --- |
| M1d-1-Duodenum | Day 1 | Duodenum | 56,304 | 1,641,820 |
| M1d-2-Duodenum |  |  | 57,547 |  |
| M1d-3-Duodenum |  |  | 38,187 |  |
| M1d-4-Duodenum |  |  | 45,081 |  |
| M1d-5-Duodenum |  |  | 64,103 |  |
| M1d-1-Jejunum |  | Jejunum | 57,498 |  |
| M1d-2-Jejunum |  |  | 58,240 |  |
| M1d-3-Jejunum |  |  | 45,186 |  |
| M1d-4-Jejunum |  |  | 64,303 |  |
| M1d-5-Jejunum |  |  | 59,671 |  |
| M1d-1-Ileum |  | Ileum | 63,839 |  |
| M1d-2-Ileum |  |  | 56,919 |  |
| M1d-3-Ileum |  |  | 61,789 |  |
| M1d-4-Ileum |  |  | 49,540 |  |
| M1d-5-Ileum |  |  | 59,177 |  |
| M1d-1-Caecum |  | Caecum | 55,929 |  |
| M1d-2-Caecum |  |  | 63,157 |  |
| M1d-3-Caecum |  |  | 61,660 |  |
| M1d-4-Caecum |  |  | 57,657 |  |
| M1d-1-Colon |  | Colon | 58,630 |  |
| M1d-2-Colon |  |  | 41,563 |  |
| M1d-3-Colon |  |  | 59,093 |  |
| M1d-4-Colon |  |  | 57,443 |  |
| M1d-5-Colon |  |  | 63,581 |  |
| M1d-1-Rectum |  | Rectum | 56,567 |  |
| M1d-2-Rectum |  |  | 60,959 |  |
| M1d-3-Rectum |  |  | 55,769 |  |
| M1d-4-Rectum |  |  | 57,229 |  |
| M1d-5-Rectum |  |  | 55,199 |  |
| M7d-1-Duodenum | Day 7 | Duodenum | 60,150 | 1,545,878 |
| M7d-3-Duodenum |  |  | 54,515 |  |
| M7d-4-Duodenum |  |  | 60,796 |  |
| M7d-5-Duodenum |  |  | 62,657 |  |
| M7d-1-Jejunum |  | Jejunum | 58,887 |  |
| M7d-2-Jejunum |  |  | 62,712 |  |
| M7d-4-Jejunum |  |  | 55,787 |  |
| M7d-5-Jejunum |  |  | 46,084 |  |
| M7d-1-Ileum |  | Ileum | 55,557 |  |
| M7d-2-Ileum |  |  | 58,843 |  |
| M7d-3-Ileum |  |  | 58,834 |  |
| M7d-4-Ileum |  |  | 55,226 |  |
| M7d-5-Ileum |  |  | 56,316 |  |
| M7d-1-Caecum |  | Caecum | 55,276 |  |
| M7d-2-Caecum |  |  | 59,212 |  |
| M7d-3-Caecum |  |  | 56,766 |  |
| M7d-4-Caecum |  |  | 61,222 |  |
| M7d-5-Caecum |  |  | 57,318 |  |
| M7d-2-Colon |  | Colon | 56,667 |  |
| M7d-3-Colon |  |  | 59,695 |  |
| M7d-4-Colon |  |  | 55,466 |  |
| M7d-5-Colon |  |  | 61,518 |  |
| M7d-1-Rectum |  | Rectum | 61,739 |  |
| M7d-2-Rectum |  |  | 55,936 |  |
| M7d-3-Rectum |  |  | 39,992 |  |
| M7d-4-Rectum |  |  | 55,199 |  |
| M7d-5-Rectum |  |  | 63,508 |  |
| M14d-1-Duodenum | Day 14 | Duodenum | 63,292 | 1,741,195 |
| M14d-2-Duodenum |  |  | 59,305 |  |
| M14d-3-Duodenum |  |  | 55,616 |  |
| M14d-4-Duodenum |  |  | 61,880 |  |
| M14d-5-Duodenum |  |  | 57,910 |  |
| M14d-2-Jejunum |  | Jejunum | 62,622 |  |
| M14d-3-Jejunum |  |  | 58,392 |  |
| M14d-4-Jejunum |  |  | 63,883 |  |
| M14d-5-Jejunum |  |  | 64,728 |  |
| M14d-1-Jejunum |  |  | 58,354 |  |
| M14d-2-Ileum |  | Ileum | 59,106 |  |
| M14d-3-Ileum |  |  | 55,713 |  |
| M14d-4-Ileum |  |  | 62,762 |  |
| M14d-5-Ileum |  |  | 62,940 |  |
| M14d-1-Caecum |  | Caecum | 59,694 |  |
| M14d-2-Caecum |  |  | 63,039 |  |
| M14d-3-Caecum |  |  | 59,256 |  |
| M14d-4-Caecum |  |  | 61,035 |  |
| M14d-5-Caecum |  |  | 56,156 |  |
| M14d-1-Colon |  | Colon | 54,484 |  |
| M14d-2-Colon |  |  | 55,221 |  |
| M14d-3-Colon |  |  | 57,624 |  |
| M14d-4-Colon |  |  | 63,392 |  |
| M14d-5-Colon |  |  | 64,410 |  |
| M14d-1-Rectum |  | Rectum | 62,807 |  |
| M14d-2-Rectum |  |  | 62,894 |  |
| M14d-3-Rectum |  |  | 60,328 |  |
| M14d-4-Rectum |  |  | 55,293 |  |
| M14d-5-Rectum |  |  | 59,059 |  |
| M21d-1-Duodenum | Day 21 | Duodenum | 55,551 | 1,667,320 |
| M21d-2-Duodenum |  |  | 64,158 |  |
| M21d-3-Duodenum |  |  | 63,655 |  |
| M21d-4-Duodenum |  |  | 47,785 |  |
| M21d-5-Duodenum |  |  | 62,516 |  |
| M21d-2-Jejunum |  | Jejunum | 63,361 |  |
| M21d-3-Jejunum |  |  | 53,607 |  |
| M21d-4-Jejunum |  |  | 62,869 |  |
| M21d-5-Jejunum |  |  | 57,414 |  |
| M21d-1-Ileum |  | Ileum | 55,252 |  |
| M21d-2-Ileum |  |  | 56,145 |  |
| M21d-3-Ileum |  |  | 62,099 |  |
| M21d-4-Ileum |  |  | 59,060 |  |
| M21d-1-Caecum |  | Caecum | 52,095 |  |
| M21d-2-Caecum |  |  | 64,723 |  |
| M21d-3-Caecum |  |  | 58,795 |  |
| M21d-4-Caecum |  |  | 59,552 |  |
| M21d-5-Caecum |  |  | 64,644 |  |
| M21d-1-Colon |  | Colon | 58,648 |  |
| M21d-2-Colon |  |  | 57,272 |  |
| M21d-3-Colon |  |  | 61,160 |  |
| M21d-4-Colon |  |  | 58,919 |  |
| M21d-5-Colon |  |  | 59,494 |  |
| M21d-1-Rectum |  | Rectum | 61,598 |  |
| M21d-2-Rectum |  |  | 59,861 |  |
| M21d-3-Rectum |  |  | 59,734 |  |
| M21d-4-Rectum |  |  | 63,816 |  |
| M21d-5-Rectum |  |  | 63,537 |  |
| M28d-1-Duodenum | Day 28 | Duodenum | 61,608 | 1,642,921 |
| M28d-2-Duodenum |  |  | 64,741 |  |
| M28d-3-Duodenum |  |  | 62,214 |  |
| M28d-4-Duodenum |  |  | 57,188 |  |
| M28d-5-Duodenum |  |  | 56,188 |  |
| M28d-1-Jejunum |  | Jejunum | 57,444 |  |
| M28d-2-Jejunum |  |  | 47,684 |  |
| M28d-3-Jejunum |  |  | 64,508 |  |
| M28d-4-Jejunum |  |  | 61,349 |  |
| M28d-5-Jejunum |  |  | 56,295 |  |
| M28d-1-Ileum |  | Ileum | 58,927 |  |
| M28d-3-Ileum |  |  | 47,673 |  |
| M28d-4-Ileum |  |  | 59,897 |  |
| M28d-5-Ileum |  |  | 58,795 |  |
| M28d-1-Caecum |  | Caecum | 56,465 |  |
| M28d-2-Caecum |  |  | 64,134 |  |
| M28d-3-Caecum |  |  | 62,941 |  |
| M28d-4-Caecum |  |  | 56,469 |  |
| M28d-5-Caecum |  |  | 62,918 |  |
| M28d-1-Colon |  | Colon | 58,205 |  |
| M28d-2-Colon |  |  | 55,517 |  |
| M28d-4-Colon |  |  | 62,017 |  |
| M28d-5-Colon |  |  | 59,275 |  |
| M28d-1-Rectum |  | Rectum | 62,950 |  |
| M28d-2-Rectum |  |  | 45,523 |  |
| M28d-3-Rectum |  |  | 55,130 |  |
| M28d-4-Rectum |  |  | 63,318 |  |
| M28d-5-Rectum |  |  | 63,548 |  |
| M35d-1-Duodenum | Day 35 | Duodenum | 61,960 | 1,778,906 |
| M35d-2-Duodenum |  |  | 58,167 |  |
| M35d-3-Duodenum |  |  | 56,722 |  |
| M35d-4-Duodenum |  |  | 61,814 |  |
| M35d-5-Duodenum |  |  | 54,091 |  |
| M35d-1-Jejunum |  | Jejunum | 58,890 |  |
| M35d-2-Jejunum |  |  | 60,203 |  |
| M35d-3-Jejunum |  |  | 61,374 |  |
| M35d-4-Jejunum |  |  | 62,798 |  |
| M35d-5-Jejunum |  |  | 54,918 |  |
| M35d-1-Ileum |  | Ileum | 57,210 |  |
| M35d-2-Ileum |  |  | 61,567 |  |
| M35d-3-Ileum |  |  | 58,143 |  |
| M35d-4-Ileum |  |  | 56,967 |  |
| M35d-5-Ileum |  |  | 64,953 |  |
| M35d-1-Caecum |  | Caecum | 64,727 |  |
| M35d-2-Caecum |  |  | 55,601 |  |
| M35d-3-Caecum |  |  | 57,036 |  |
| M35d-4-Caecum |  |  | 55,152 |  |
| M35d-5-Caecum |  |  | 61,297 |  |
| M35d-1-Colon |  | Colon | 58,976 |  |
| M35d-2-Colon |  |  | 48,945 |  |
| M35d-3-Colon |  |  | 60,100 |  |
| M35d-4-Colon |  |  | 64,294 |  |
| M35d-5-Colon |  |  | 63,222 |  |
| M35d-1-Rectum |  | Rectum | 61,231 |  |
| M35d-2-Rectum |  |  | 58,761 |  |
| M35d-3-Rectum |  |  | 57,808 |  |
| M35d-4-Rectum |  |  | 62,221 |  |
| M35d-5-Rectum |  |  | 59,758 |  |
| M120d-1-Duodenum | Day 120 | Duodenum | 56,200 | 1,704,472 |
| M120d-2-Duodenum |  |  | 57,447 |  |
| M120d-3-Duodenum |  |  | 59,258 |  |
| M120d-4-Duodenum |  |  | 55,460 |  |
| M120d-1-Jejunum |  | Jejunum | 57,247 |  |
| M120d-2-Jejunum |  |  | 55,232 |  |
| M120d-3-Jejunum |  |  | 63,858 |  |
| M120d-4-Jejunum |  |  | 61,050 |  |
| M120d-5-Jejunum |  |  | 62,037 |  |
| M120d-1-Ileum |  | Ileum | 64,178 |  |
| M120d-2-Ileum |  |  | 59,823 |  |
| M120d-3-Ileum |  |  | 62,117 |  |
| M120d-4-Ileum |  |  | 62,830 |  |
| M120d-5-Ileum |  |  | 62,895 |  |
| M120d-1-Caecum |  | Caecum | 62,848 |  |
| M120d-2-Caecum |  |  | 62,102 |  |
| M120d-3-Caecum |  |  | 62,359 |  |
| M120d-4-Caecum |  |  | 51,557 |  |
| M120d-5-Caecum |  |  | 61,379 |  |
| M120d-1-Colon |  | Colon | 55,691 |  |
| M120d-2-Colon |  |  | 63,501 |  |
| M120d-3-Colon |  |  | 55,500 |  |
| M120d-4-Colon |  |  | 55,583 |  |
| M120d-5-Colon |  |  | 63,937 |  |
| M120d-1-Rectum |  | Rectum | 58,980 |  |
| M120d-2-Rectum |  |  | 46,665 |  |
| M120d-3-Rectum |  |  | 50,147 |  |
| M120d-4-Rectum |  |  | 55,072 |  |
| M120d-5-Rectum |  |  | 59,519 |  |
| M180d-1-Duodenum | Day 180 | Duodenum | 55,544 | 1,783,935 |
| M180d-2-Duodenum |  |  | 62,960 |  |
| M180d-3-Duodenum |  |  | 57,609 |  |
| M180d-4-Duodenum |  |  | 60,601 |  |
| M180d-5-Duodenum |  |  | 54,620 |  |
| M180d-1-Jejunum |  | Jejunum | 61,363 |  |
| M180d-2-Jejunum |  |  | 62,972 |  |
| M180d-3-Jejunum |  |  | 64,008 |  |
| M180d-4-Jejunum |  |  | 59,510 |  |
| M180d-5-Jejunum |  |  | 57,576 |  |
| M180d-1-Ileum |  | Ileum | 64,262 |  |
| M180d-2-Ileum |  |  | 55,615 |  |
| M180d-3-Ileum |  |  | 56,257 |  |
| M180d-4-Ileum |  |  | 49,273 |  |
| M180d-5-Ileum |  |  | 55,965 |  |
| M180d-1-Caecum |  | Caecum | 60,481 |  |
| M180d-2-Caecum |  |  | 62,479 |  |
| M180d-3-Caecum |  |  | 63,591 |  |
| M180d-4-Caecum |  |  | 61,854 |  |
| M180d-5-Caecum |  |  | 64,392 |  |
| M180d-1-Colon |  | Colon | 63,287 |  |
| M180d-2-Colon |  |  | 59,503 |  |
| M180d-3-Colon |  |  | 57,941 |  |
| M180d-4-Colon |  |  | 62,312 |  |
| M180d-5-Colon |  |  | 59,011 |  |
| M180d-1-Rectum |  | Rectum | 56,856 |  |
| M180d-2-Rectum |  |  | 58,515 |  |
| M180d-3-Rectum |  |  | 55,906 |  |
| M180d-4-Rectum |  |  | 61,126 |  |
| M180d-5-Rectum |  |  | 58,546 |  |
| Sum |  |  | 13,506,447 |  |

Table S3

The numbers of OTUs in each sample among different intestinal segments

| Sample name | Intestinal segments | OTUs | Average OTUs |
| --- | --- | --- | --- |
| M1d-1-Duodenum | Duodenum | 410 | 424.1 |
| M1d-2-Duodenum |  | 483 |  |
| M1d-3-Duodenum |  | 428 |  |
| M1d-4-Duodenum |  | 533 |  |
| M1d-5-Duodenum |  | 427 |  |
| M7d-1-Duodenum |  | 516 |  |
| M7d-3-Duodenum |  | 566 |  |
| M7d-4-Duodenum |  | 566 |  |
| M7d-5-Duodenum |  | 499 |  |
| M14d-1-Duodenum |  | 285 |  |
| M14d-2-Duodenum |  | 454 |  |
| M14d-3-Duodenum |  | 332 |  |
| M14d-4-Duodenum |  | 314 |  |
| M14d-5-Duodenum |  | 229 |  |
| M21d-1-Duodenum |  | 338 |  |
| M21d-2-Duodenum |  | 500 |  |
| M21d-3-Duodenum |  | 497 |  |
| M21d-4-Duodenum |  | 390 |  |
| M21d-5-Duodenum |  | 545 |  |
| M28d-1-Duodenum |  | 190 |  |
| M28d-2-Duodenum |  | 383 |  |
| M28d-3-Duodenum |  | 242 |  |
| M28d-4-Duodenum |  | 222 |  |
| M28d-5-Duodenum |  | 460 |  |
| M35d-1-Duodenum |  | 266 |  |
| M35d-2-Duodenum |  | 347 |  |
| M35d-3-Duodenum |  | 447 |  |
| M35d-4-Duodenum |  | 466 |  |
| M35d-5-Duodenum |  | 314 |  |
| M120d-1-Duodenum |  | 441 |  |
| M120d-2-Duodenum |  | 735 |  |
| M120d-3-Duodenum |  | 363 |  |
| M120d-4-Duodenum |  | 297 |  |
| M180d-1-Duodenum |  | 551 |  |
| M180d-2-Duodenum |  | 347 |  |
| M180d-3-Duodenum |  | 632 |  |
| M180d-4-Duodenum |  | 267 |  |
| M180d-5-Duodenum |  | 835 |  |
| M1d-1-Jejunum | Jejunum | 397 | 415.7 |
| M1d-2-Jejunum |  | 349 |  |
| M1d-3-Jejunum |  | 431 |  |
| M1d-4-Jejunum |  | 339 |  |
| M1d-5-Jejunum |  | 514 |  |
| M7d-1-Jejunum |  | 346 |  |
| M7d-2-Jejunum |  | 1159 |  |
| M7d-4-Jejunum |  | 576 |  |
| M7d-5-Jejunum |  | 366 |  |
| M14d-2-Jejunum |  | 288 |  |
| M14d-3-Jejunum |  | 275 |  |
| M14d-4-Jejunum |  | 117 |  |
| M14d-5-Jejunum |  | 233 |  |
| M14d-1-Jejunum |  | 301 |  |
| M21d-2-Jejunum |  | 208 |  |
| M21d-3-Jejunum |  | 363 |  |
| M21d-4-Jejunum |  | 237 |  |
| M21d-5-Jejunum |  | 283 |  |
| M28d-1-Jejunum |  | 192 |  |
| M28d-2-Jejunum |  | 845 |  |
| M28d-3-Jejunum |  | 1006 |  |
| M28d-4-Jejunum |  | 337 |  |
| M28d-5-Jejunum |  | 508 |  |
| M35d-1-Jejunum |  | 457 |  |
| M35d-2-Jejunum |  | 385 |  |
| M35d-3-Jejunum |  | 396 |  |
| M35d-4-Jejunum |  | 171 |  |
| M35d-5-Jejunum |  | 851 |  |
| M120d-1-Jejunum |  | 255 |  |
| M120d-2-Jejunum |  | 403 |  |
| M120d-3-Jejunum |  | 349 |  |
| M120d-4-Jejunum |  | 445 |  |
| M120d-5-Jejunum |  | 354 |  |
| M180d-1-Jejunum |  | 554 |  |
| M180d-2-Jejunum |  | 234 |  |
| M180d-3-Jejunum |  | 553 |  |
| M180d-4-Jejunum |  | 340 |  |
| M180d-5-Jejunum |  | 381 |  |
| M1d-1-Ileum | Ileum | 312 | 357.3 |
| M1d-2-Ileum |  | 427 |  |
| M1d-3-Ileum |  | 459 |  |
| M1d-4-Ileum |  | 384 |  |
| M1d-5-Ileum |  | 376 |  |
| M7d-1-Ileum |  | 310 |  |
| M7d-2-Ileum |  | 355 |  |
| M7d-3-Ileum |  | 217 |  |
| M7d-4-Ileum |  | 432 |  |
| M7d-5-Ileum |  | 502 |  |
| M14d-2-Ileum |  | 284 |  |
| M14d-3-Ileum |  | 300 |  |
| M14d-4-Ileum |  | 90 |  |
| M14d-5-Ileum |  | 333 |  |
| M21d-1-Ileum |  | 224 |  |
| M21d-2-Ileum |  | 326 |  |
| M21d-3-Ileum |  | 254 |  |
| M21d-4-Ileum |  | 424 |  |
| M28d-1-Ileum |  | 104 |  |
| M28d-3-Ileum |  | 222 |  |
| M28d-4-Ileum |  | 250 |  |
| M28d-5-Ileum |  | 207 |  |
| M35d-1-Ileum |  | 184 |  |
| M35d-2-Ileum |  | 314 |  |
| M35d-3-Ileum |  | 353 |  |
| M35d-4-Ileum |  | 255 |  |
| M35d-5-Ileum |  | 386 |  |
| M120d-1-Ileum |  | 196 |  |
| M120d-2-Ileum |  | 202 |  |
| M120d-3-Ileum |  | 519 |  |
| M120d-4-Ileum |  | 443 |  |
| M120d-5-Ileum |  | 409 |  |
| M180d-1-Ileum |  | 687 |  |
| M180d-2-Ileum |  | 894 |  |
| M180d-3-Ileum |  | 513 |  |
| M180d-4-Ileum |  | 467 |  |
| M180d-5-Ileum |  | 606 |  |
| M1d-1-Caecum | Caecum | 356 | 526.0 |
| M1d-2-Caecum |  | 818 |  |
| M1d-3-Caecum |  | 499 |  |
| M1d-4-Caecum |  | 423 |  |
| M7d-1-Caecum |  | 308 |  |
| M7d-2-Caecum |  | 552 |  |
| M7d-3-Caecum |  | 457 |  |
| M7d-4-Caecum |  | 472 |  |
| M7d-5-Caecum |  | 363 |  |
| M14d-1-Caecum |  | 291 |  |
| M14d-2-Caecum |  | 609 |  |
| M14d-3-Caecum |  | 241 |  |
| M14d-4-Caecum |  | 323 |  |
| M14d-5-Caecum |  | 513 |  |
| M21d-1-Caecum |  | 596 |  |
| M21d-2-Caecum |  | 339 |  |
| M21d-3-Caecum |  | 439 |  |
| M21d-4-Caecum |  | 561 |  |
| M21d-5-Caecum |  | 366 |  |
| M28d-1-Caecum |  | 452 |  |
| M28d-2-Caecum |  | 422 |  |
| M28d-3-Caecum |  | 710 |  |
| M28d-4-Caecum |  | 327 |  |
| M28d-5-Caecum |  | 432 |  |
| M35d-1-Caecum |  | 439 |  |
| M35d-2-Caecum |  | 748 |  |
| M35d-3-Caecum |  | 365 |  |
| M35d-4-Caecum |  | 405 |  |
| M35d-5-Caecum |  | 661 |  |
| M120d-1-Caecum |  | 710 |  |
| M120d-2-Caecum |  | 544 |  |
| M120d-3-Caecum |  | 547 |  |
| M120d-4-Caecum |  | 535 |  |
| M120d-5-Caecum |  | 539 |  |
| M180d-1-Caecum |  | 918 |  |
| M180d-2-Caecum |  | 1068 |  |
| M180d-3-Caecum |  | 937 |  |
| M180d-4-Caecum |  | 467 |  |
| M180d-5-Caecum |  | 763 |  |
| M1d-1-Colon | Colon | 266 | 537.6 |
| M1d-2-Colon |  | 321 |  |
| M1d-3-Colon |  | 438 |  |
| M1d-4-Colon |  | 345 |  |
| M1d-5-Colon |  | 492 |  |
| M7d-2-Colon |  | 499 |  |
| M7d-3-Colon |  | 469 |  |
| M7d-4-Colon |  | 286 |  |
| M7d-5-Colon |  | 367 |  |
| M14d-1-Colon |  | 278 |  |
| M14d-2-Colon |  | 618 |  |
| M14d-3-Colon |  | 268 |  |
| M14d-4-Colon |  | 278 |  |
| M14d-5-Colon |  | 499 |  |
| M21d-1-Colon |  | 575 |  |
| M21d-2-Colon |  | 584 |  |
| M21d-3-Colon |  | 603 |  |
| M21d-4-Colon |  | 586 |  |
| M21d-5-Colon |  | 393 |  |
| M28d-1-Colon |  | 500 |  |
| M28d-2-Colon |  | 406 |  |
| M28d-4-Colon |  | 464 |  |
| M28d-5-Colon |  | 505 |  |
| M35d-1-Colon |  | 637 |  |
| M35d-2-Colon |  | 484 |  |
| M35d-3-Colon |  | 318 |  |
| M35d-4-Colon |  | 497 |  |
| M35d-5-Colon |  | 626 |  |
| M120d-1-Colon |  | 991 |  |
| M120d-2-Colon |  | 618 |  |
| M120d-3-Colon |  | 715 |  |
| M120d-4-Colon |  | 503 |  |
| M120d-5-Colon |  | 588 |  |
| M180d-1-Colon |  | 1049 |  |
| M180d-2-Colon |  | 1059 |  |
| M180d-3-Colon |  | 1101 |  |
| M180d-4-Colon |  | 416 |  |
| M180d-5-Colon |  | 786 |  |
| M1d-1-Rectum | Rectum | 334 | 520.5 |
| M1d-2-Rectum |  | 432 |  |
| M1d-3-Rectum |  | 357 |  |
| M1d-4-Rectum |  | 359 |  |
| M1d-5-Rectum |  | 441 |  |
| M7d-1-Rectum |  | 343 |  |
| M7d-2-Rectum |  | 865 |  |
| M7d-3-Rectum |  | 430 |  |
| M7d-4-Rectum |  | 300 |  |
| M7d-5-Rectum |  | 347 |  |
| M14d-1-Rectum |  | 293 |  |
| M14d-2-Rectum |  | 620 |  |
| M14d-3-Rectum |  | 324 |  |
| M14d-4-Rectum |  | 237 |  |
| M14d-5-Rectum |  | 520 |  |
| M21d-1-Rectum |  | 598 |  |
| M21d-2-Rectum |  | 386 |  |
| M21d-3-Rectum |  | 510 |  |
| M21d-4-Rectum |  | 591 |  |
| M21d-5-Rectum |  | 361 |  |
| M28d-1-Rectum |  | 457 |  |
| M28d-2-Rectum |  | 381 |  |
| M28d-3-Rectum |  | 688 |  |
| M28d-4-Rectum |  | 348 |  |
| M28d-5-Rectum |  | 417 |  |
| M35d-1-Rectum |  | 673 |  |
| M35d-2-Rectum |  | 588 |  |
| M35d-3-Rectum |  | 351 |  |
| M35d-4-Rectum |  | 426 |  |
| M35d-5-Rectum |  | 672 |  |
| M120d-1-Rectum |  | 571 |  |
| M120d-2-Rectum |  | 598 |  |
| M120d-3-Rectum |  | 696 |  |
| M120d-4-Rectum |  | 689 |  |
| M120d-5-Rectum |  | 637 |  |
| M180d-1-Rectum |  | 1019 |  |
| M180d-2-Rectum |  | 687 |  |
| M180d-3-Rectum |  | 921 |  |
| M180d-4-Rectum |  | 480 |  |
| M180d-5-Rectum |  | 874 |  |

Table S4

The numbers of OTUs in each sample during different growth stages

| Sample name | Growth stages | Intestinal segments | OTUs | Average OTUs |
| --- | --- | --- | --- | --- |
| M1d-1-Duodenum | Day 1 | Duodenum | 410 | 456.2 |
| M1d-2-Duodenum |  |  | 483 |  |
| M1d-3-Duodenum |  |  | 428 |  |
| M1d-4-Duodenum |  |  | 533 |  |
| M1d-5-Duodenum |  |  | 427 |  |
| M1d-1-Jejunum |  | Jejunum | 397 | 406 |
| M1d-2-Jejunum |  |  | 349 |  |
| M1d-3-Jejunum |  |  | 431 |  |
| M1d-4-Jejunum |  |  | 339 |  |
| M1d-5-Jejunum |  |  | 514 |  |
| M1d-1-Ileum |  | Ileum | 312 | 391.6 |
| M1d-2-Ileum |  |  | 427 |  |
| M1d-3-Ileum |  |  | 459 |  |
| M1d-4-Ileum |  |  | 384 |  |
| M1d-5-Ileum |  |  | 376 |  |
| M1d-1-Caecum |  | Caecum | 356 | 389.5 |
| M1d-2-Caecum |  |  | 818 |  |
| M1d-3-Caecum |  |  | 499 |  |
| M1d-4-Caecum |  |  | 423 |  |
| M1d-1-Colon |  | Colon | 266 | 372.4 |
| M1d-2-Colon |  |  | 321 |  |
| M1d-3-Colon |  |  | 438 |  |
| M1d-4-Colon |  |  | 345 |  |
| M1d-5-Colon |  |  | 492 |  |
| M1d-1-Rectum |  | Rectum | 334 | 384.6 |
| M1d-2-Rectum |  |  | 432 |  |
| M1d-3-Rectum |  |  | 357 |  |
| M1d-4-Rectum |  |  | 359 |  |
| M1d-5-Rectum |  |  | 441 |  |
| M7d-1-Duodenum | Day 7 | Duodenum | 516 | 507.5 |
| M7d-3-Duodenum |  |  | 566 |  |
| M7d-4-Duodenum |  |  | 566 |  |
| M7d-5-Duodenum |  |  | 499 |  |
| M7d-1-Jejunum |  | Jejunum | 346 | 356 |
| M7d-2-Jejunum |  |  | 1159 |  |
| M7d-4-Jejunum |  |  | 576 |  |
| M7d-5-Jejunum |  |  | 366 |  |
| M7d-1-Ileum |  | Ileum | 310 | 363.2 |
| M7d-2-Ileum |  |  | 355 |  |
| M7d-3-Ileum |  |  | 217 |  |
| M7d-4-Ileum |  |  | 432 |  |
| M7d-5-Ileum |  |  | 502 |  |
| M7d-1-Caecum |  | Caecum | 308 | 430.4 |
| M7d-2-Caecum |  |  | 552 |  |
| M7d-3-Caecum |  |  | 457 |  |
| M7d-4-Caecum |  |  | 472 |  |
| M7d-5-Caecum |  |  | 363 |  |
| M7d-2-Colon |  | Colon | 499 | 433 |
| M7d-3-Colon |  |  | 469 |  |
| M7d-4-Colon |  |  | 286 |  |
| M7d-5-Colon |  |  | 367 |  |
| M7d-1-Rectum |  | Rectum | 343 | 457 |
| M7d-2-Rectum |  |  | 865 |  |
| M7d-3-Rectum |  |  | 430 |  |
| M7d-4-Rectum |  |  | 300 |  |
| M7d-5-Rectum |  |  | 347 |  |
| M14d-1-Duodenum | Day 14 | Duodenum | 285 | 322.8 |
| M14d-2-Duodenum |  |  | 454 |  |
| M14d-3-Duodenum |  |  | 332 |  |
| M14d-4-Duodenum |  |  | 314 |  |
| M14d-5-Duodenum |  |  | 229 |  |
| M14d-2-Jejunum |  | Jejunum | 288 | 242.8 |
| M14d-3-Jejunum |  |  | 275 |  |
| M14d-4-Jejunum |  |  | 117 |  |
| M14d-5-Jejunum |  |  | 233 |  |
| M14d-1-Jejunum |  |  | 301 |  |
| M14d-2-Ileum |  | Ileum | 284 | 308.5 |
| M14d-3-Ileum |  |  | 300 |  |
| M14d-4-Ileum |  |  | 90 |  |
| M14d-5-Ileum |  |  | 333 |  |
| M14d-1-Caecum |  | Caecum | 291 | 395.4 |
| M14d-2-Caecum |  |  | 609 |  |
| M14d-3-Caecum |  |  | 241 |  |
| M14d-4-Caecum |  |  | 323 |  |
| M14d-5-Caecum |  |  | 513 |  |
| M14d-1-Colon |  | Colon | 278 | 388.2 |
| M14d-2-Colon |  |  | 618 |  |
| M14d-3-Colon |  |  | 268 |  |
| M14d-4-Colon |  |  | 278 |  |
| M14d-5-Colon |  |  | 499 |  |
| M14d-1-Rectum |  | Rectum | 293 | 398.8 |
| M14d-2-Rectum |  |  | 620 |  |
| M14d-3-Rectum |  |  | 324 |  |
| M14d-4-Rectum |  |  | 237 |  |
| M14d-5-Rectum |  |  | 520 |  |
| M21d-1-Duodenum | Day 21 | Duodenum | 338 | 454 |
| M21d-2-Duodenum |  |  | 500 |  |
| M21d-3-Duodenum |  |  | 497 |  |
| M21d-4-Duodenum |  |  | 390 |  |
| M21d-5-Duodenum |  |  | 545 |  |
| M21d-2-Jejunum |  | Jejunum | 208 | 245.5 |
| M21d-3-Jejunum |  |  | 363 |  |
| M21d-4-Jejunum |  |  | 237 |  |
| M21d-5-Jejunum |  |  | 283 |  |
| M21d-1-Ileum |  | Ileum | 224 | 324 |
| M21d-2-Ileum |  |  | 326 |  |
| M21d-3-Ileum |  |  | 254 |  |
| M21d-4-Ileum |  |  | 424 |  |
| M21d-1-Caecum |  | Caecum | 596 | 460.2 |
| M21d-2-Caecum |  |  | 339 |  |
| M21d-3-Caecum |  |  | 439 |  |
| M21d-4-Caecum |  |  | 561 |  |
| M21d-5-Caecum |  |  | 366 |  |
| M21d-1-Colon |  | Colon | 575 | 548.2 |
| M21d-2-Colon |  |  | 584 |  |
| M21d-3-Colon |  |  | 603 |  |
| M21d-4-Colon |  |  | 586 |  |
| M21d-5-Colon |  |  | 393 |  |
| M21d-1-Rectum |  | Rectum | 598 | 489.2 |
| M21d-2-Rectum |  |  | 386 |  |
| M21d-3-Rectum |  |  | 510 |  |
| M21d-4-Rectum |  |  | 591 |  |
| M21d-5-Rectum |  |  | 361 |  |
| M28d-1-Duodenum | 28 days | Duodenum | 190 | 299.4 |
| M28d-2-Duodenum |  |  | 383 |  |
| M28d-3-Duodenum |  |  | 242 |  |
| M28d-4-Duodenum |  |  | 222 |  |
| M28d-5-Duodenum |  |  | 460 |  |
| M28d-1-Jejunum |  | Jejunum | 192 | 577.6 |
| M28d-2-Jejunum |  |  | 845 |  |
| M28d-3-Jejunum |  |  | 1006 |  |
| M28d-4-Jejunum |  |  | 337 |  |
| M28d-5-Jejunum |  |  | 508 |  |
| M28d-1-Ileum |  | Ileum | 104 | 155.5 |
| M28d-3-Ileum |  |  | 222 |  |
| M28d-4-Ileum |  |  | 250 |  |
| M28d-5-Ileum |  |  | 207 |  |
| M28d-1-Caecum |  | Caecum | 452 | 468.6 |
| M28d-2-Caecum |  |  | 422 |  |
| M28d-3-Caecum |  |  | 710 |  |
| M28d-4-Caecum |  |  | 327 |  |
| M28d-5-Caecum |  |  | 432 |  |
| M28d-1-Colon |  | Colon | 500 | 502.5 |
| M28d-2-Colon |  |  | 406 |  |
| M28d-4-Colon |  |  | 464 |  |
| M28d-5-Colon |  |  | 505 |  |
| M28d-1-Rectum |  | Rectum | 457 | 458.2 |
| M28d-2-Rectum |  |  | 381 |  |
| M28d-3-Rectum |  |  | 688 |  |
| M28d-4-Rectum |  |  | 348 |  |
| M28d-5-Rectum |  |  | 417 |  |
| M35d-1-Duodenum | Day 35 | Duodenum | 266 | 368 |
| M35d-2-Duodenum |  |  | 347 |  |
| M35d-3-Duodenum |  |  | 447 |  |
| M35d-4-Duodenum |  |  | 466 |  |
| M35d-5-Duodenum |  |  | 314 |  |
| M35d-1-Jejunum |  | Jejunum | 457 | 452 |
| M35d-2-Jejunum |  |  | 385 |  |
| M35d-3-Jejunum |  |  | 396 |  |
| M35d-4-Jejunum |  |  | 171 |  |
| M35d-5-Jejunum |  |  | 851 |  |
| M35d-1-Ileum |  | Ileum | 184 | 298.4 |
| M35d-2-Ileum |  |  | 314 |  |
| M35d-3-Ileum |  |  | 353 |  |
| M35d-4-Ileum |  |  | 255 |  |
| M35d-5-Ileum |  |  | 386 |  |
| M35d-1-Caecum |  | Caecum | 439 | 523.6 |
| M35d-2-Caecum |  |  | 748 |  |
| M35d-3-Caecum |  |  | 365 |  |
| M35d-4-Caecum |  |  | 405 |  |
| M35d-5-Caecum |  |  | 661 |  |
| M35d-1-Colon |  | Colon | 637 | 512.4 |
| M35d-2-Colon |  |  | 484 |  |
| M35d-3-Colon |  |  | 318 |  |
| M35d-4-Colon |  |  | 497 |  |
| M35d-5-Colon |  |  | 626 |  |
| M35d-1-Rectum |  | Rectum | 673 | 542 |
| M35d-2-Rectum |  |  | 588 |  |
| M35d-3-Rectum |  |  | 351 |  |
| M35d-4-Rectum |  |  | 426 |  |
| M35d-5-Rectum |  |  | 672 |  |
| M120d-1-Duodenum | Day 120 | Duodenum | 441 | 369 |
| M120d-2-Duodenum |  |  | 735 |  |
| M120d-3-Duodenum |  |  | 363 |  |
| M120d-4-Duodenum |  |  | 297 |  |
| M120d-1-Jejunum |  | Jejunum | 255 | 361.2 |
| M120d-2-Jejunum |  |  | 403 |  |
| M120d-3-Jejunum |  |  | 349 |  |
| M120d-4-Jejunum |  |  | 445 |  |
| M120d-5-Jejunum |  |  | 354 |  |
| M120d-1-Ileum |  | Ileum | 196 | 353.8 |
| M120d-2-Ileum |  |  | 202 |  |
| M120d-3-Ileum |  |  | 519 |  |
| M120d-4-Ileum |  |  | 443 |  |
| M120d-5-Ileum |  |  | 409 |  |
| M120d-1-Caecum |  | Caecum | 710 | 575 |
| M120d-2-Caecum |  |  | 544 |  |
| M120d-3-Caecum |  |  | 547 |  |
| M120d-4-Caecum |  |  | 535 |  |
| M120d-5-Caecum |  |  | 539 |  |
| M120d-1-Colon |  | Colon | 991 | 683 |
| M120d-2-Colon |  |  | 618 |  |
| M120d-3-Colon |  |  | 715 |  |
| M120d-4-Colon |  |  | 503 |  |
| M120d-5-Colon |  |  | 588 |  |
| M120d-1-Rectum |  | Rectum | 571 | 638.2 |
| M120d-2-Rectum |  |  | 598 |  |
| M120d-3-Rectum |  |  | 696 |  |
| M120d-4-Rectum |  |  | 689 |  |
| M120d-5-Rectum |  |  | 637 |  |
| M180d-1-Duodenum | Day 180 | Duodenum | 551 | 526.4 |
| M180d-2-Duodenum |  |  | 347 |  |
| M180d-3-Duodenum |  |  | 632 |  |
| M180d-4-Duodenum |  |  | 267 |  |
| M180d-5-Duodenum |  |  | 835 |  |
| M180d-1-Jejunum |  | Jejunum | 554 | 412.4 |
| M180d-2-Jejunum |  |  | 234 |  |
| M180d-3-Jejunum |  |  | 553 |  |
| M180d-4-Jejunum |  |  | 340 |  |
| M180d-5-Jejunum |  |  | 381 |  |
| M180d-1-Ileum |  | Ileum | 687 | 633.4 |
| M180d-2-Ileum |  |  | 894 |  |
| M180d-3-Ileum |  |  | 513 |  |
| M180d-4-Ileum |  |  | 467 |  |
| M180d-5-Ileum |  |  | 606 |  |
| M180d-1-Caecum |  | Caecum | 918 | 830.6 |
| M180d-2-Caecum |  |  | 1068 |  |
| M180d-3-Caecum |  |  | 937 |  |
| M180d-4-Caecum |  |  | 467 |  |
| M180d-5-Caecum |  |  | 763 |  |
| M180d-1-Colon |  | Colon | 1049 | 882.2 |
| M180d-2-Colon |  |  | 1059 |  |
| M180d-3-Colon |  |  | 1101 |  |
| M180d-4-Colon |  |  | 416 |  |
| M180d-5-Colon |  |  | 786 |  |
| M180d-1-Rectum |  | Rectum | 1019 | 796.2 |
| M180d-2-Rectum |  |  | 687 |  |
| M180d-3-Rectum |  |  | 921 |  |
| M180d-4-Rectum |  |  | 480 |  |
| M180d-5-Rectum |  |  | 874 |  |


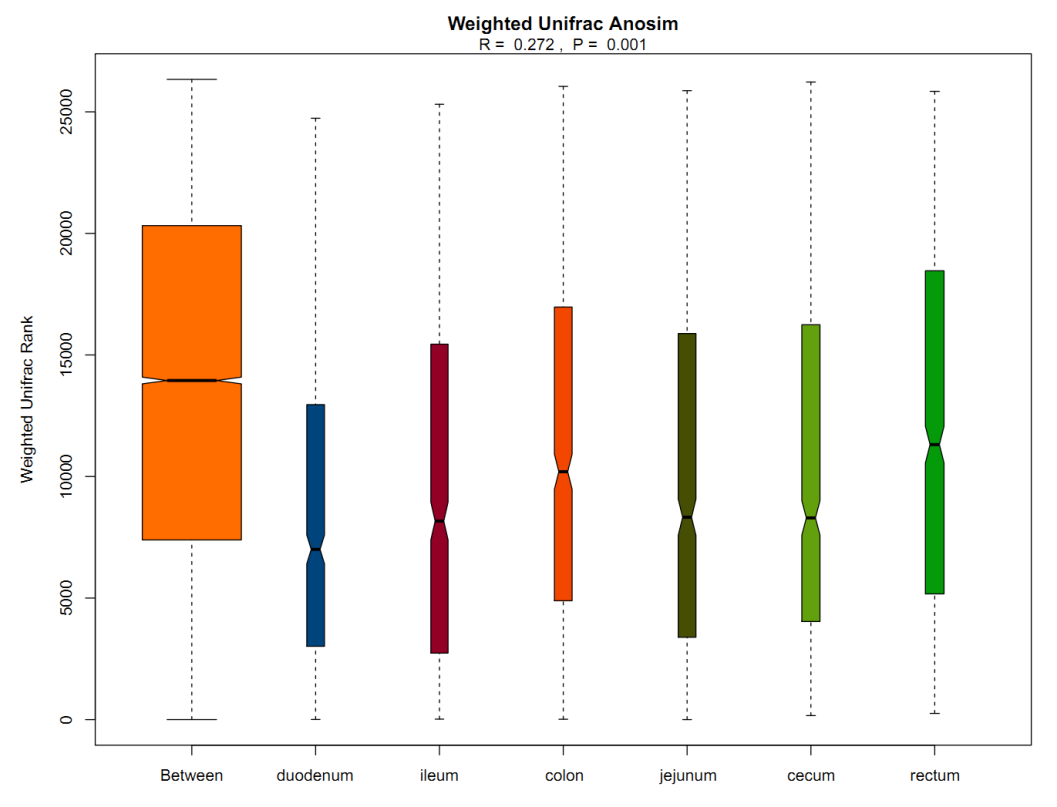


**Figure S1.** The microbiome differences among the six intestinal segments based on Weighted Unifrac distances.

**
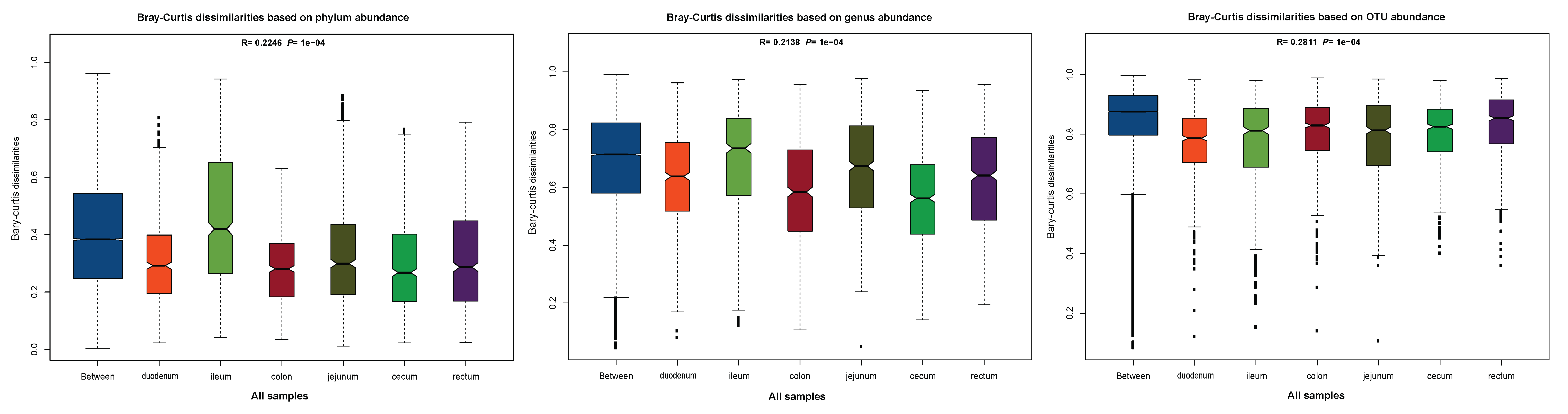
**

**Figure S2.** Bray-Curtis dissimilarities was calculated for the six intestinal segments based on the phylum abundance (a), genus abundance (b) and OTU abundance (c).


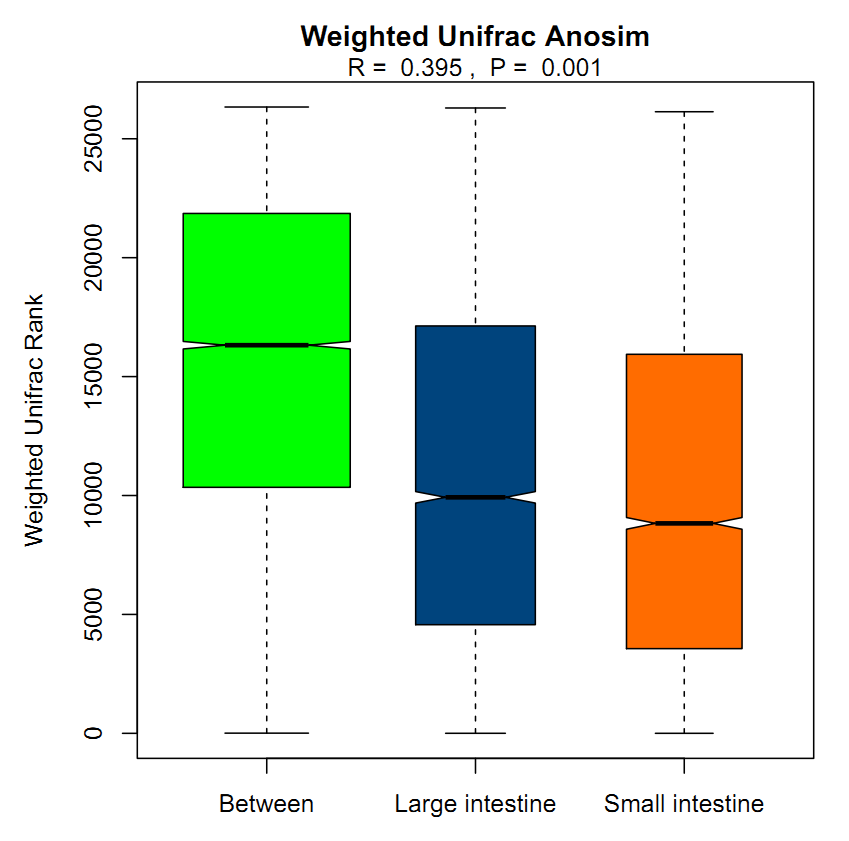


**Figure S3.** The microbiome difference between the small intestine and large intestine based on Weighted Unifrac distances.

**
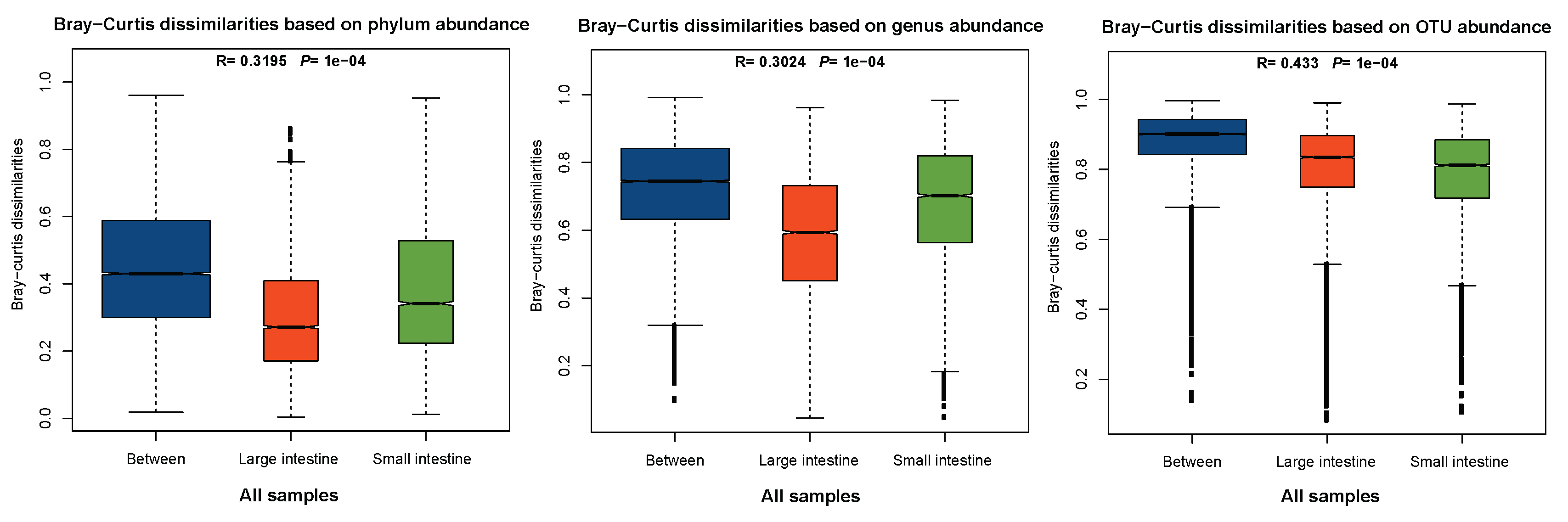
**

**Figure S4.** Bray-Curtis dissimilarities was calculated for the small intestine and large intestine based on the phylum abundance (a), genus abundance (b) and OTU abundance (c).


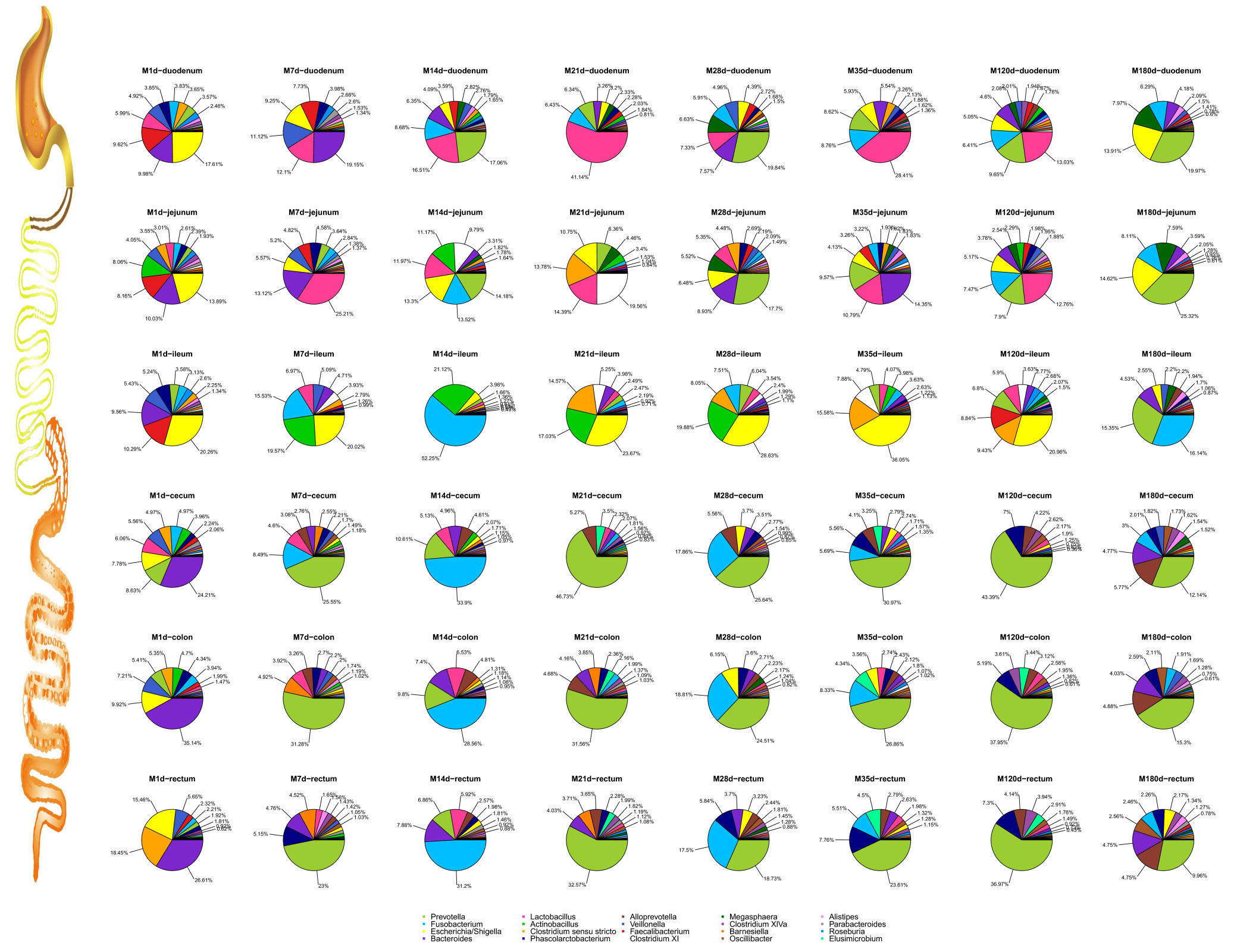


**Figure S5.** Gut microbiota composition among different intestinal segments during different growth stages at the genus level. The results are arranged into six rows that correspond to the six intestinal segments (i.e., the duodenum, jejunum, ileum, cecum, colon and rectum, from top to bottom) and eight columns that correspond to eight time points (i.e., postnatal days 1, 7, 14, 21, 28, 35, 120 and 180, from left to right). Each pie chart indicates the composition of the gut microbiome in a certain intestinal segment at a given time point. Different colors represent different bacterial genus. The percentages represent the relative abundance of individual genera.

.

**
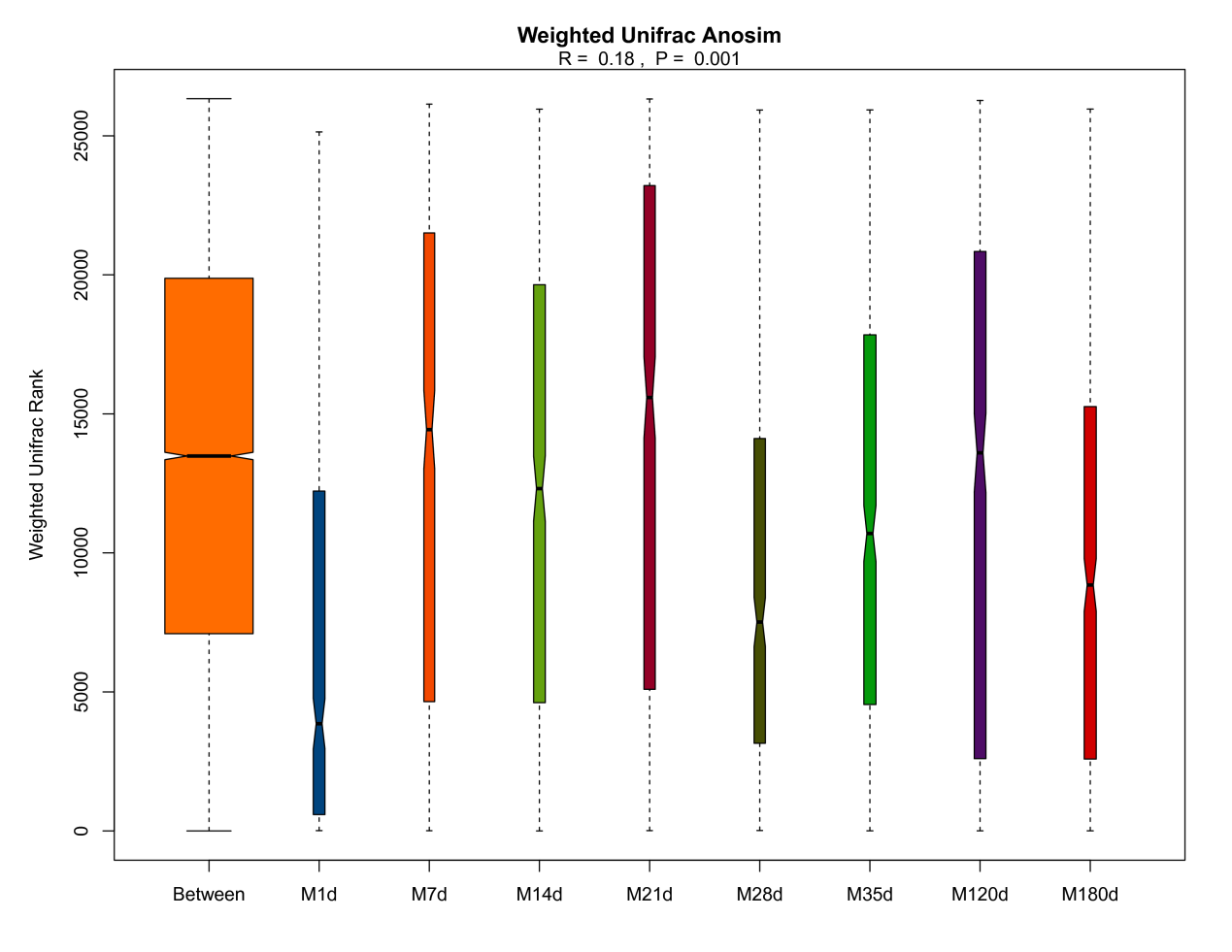
**

**Figure S6.** The microbiome differences among the eight postnatal intervals based on Weighted Unifrac distances.

**
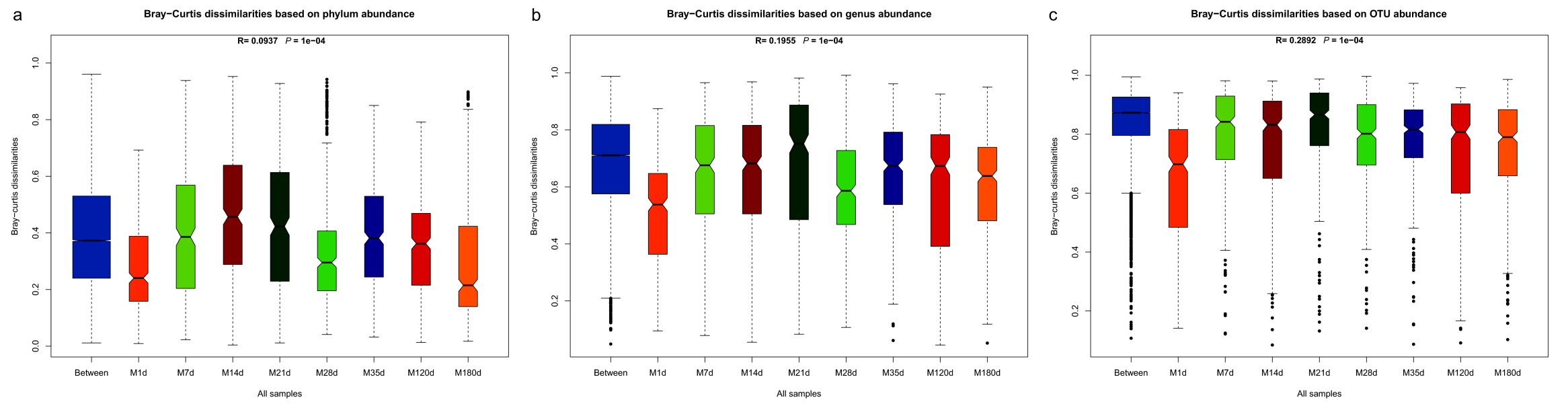
**

**Figure S7.** Bray-Curtis dissimilarities was calculated for the the eight postnatal intervals based on the phylum abundance (a), genus abundance (b) and OTU abundance (c).


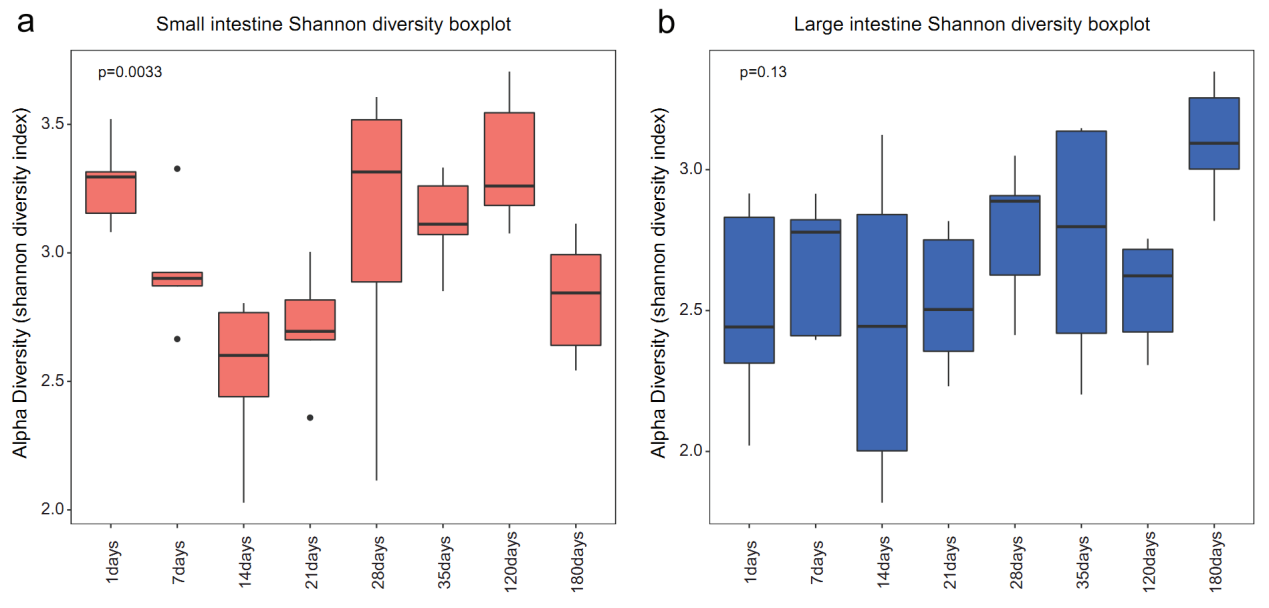


**Figure S8.** The Shannon diversity indices of the small intestine (a) and large intestine (b). The boxes represent the interquartile range (IQR); the lines in boxes indicate the median values. The whiskers represent the lowest and highest values within 1.5 interquartile range (IQR) from the first and third quartiles. The black dots denote the outliers.


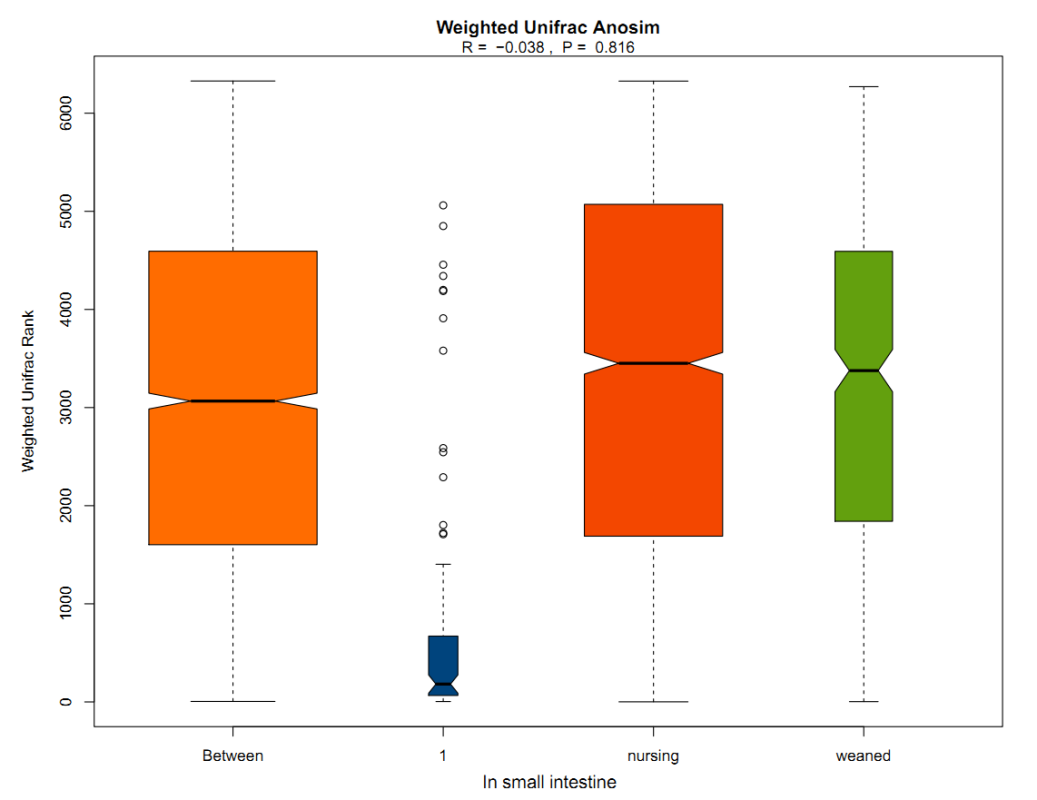


**Figure S9.** The Weighted Unifrac distances revealed that there was no significant difference among day 1, nursing period and weaning period in the small intestine (R = -0.038, *p* = 0.816).

**
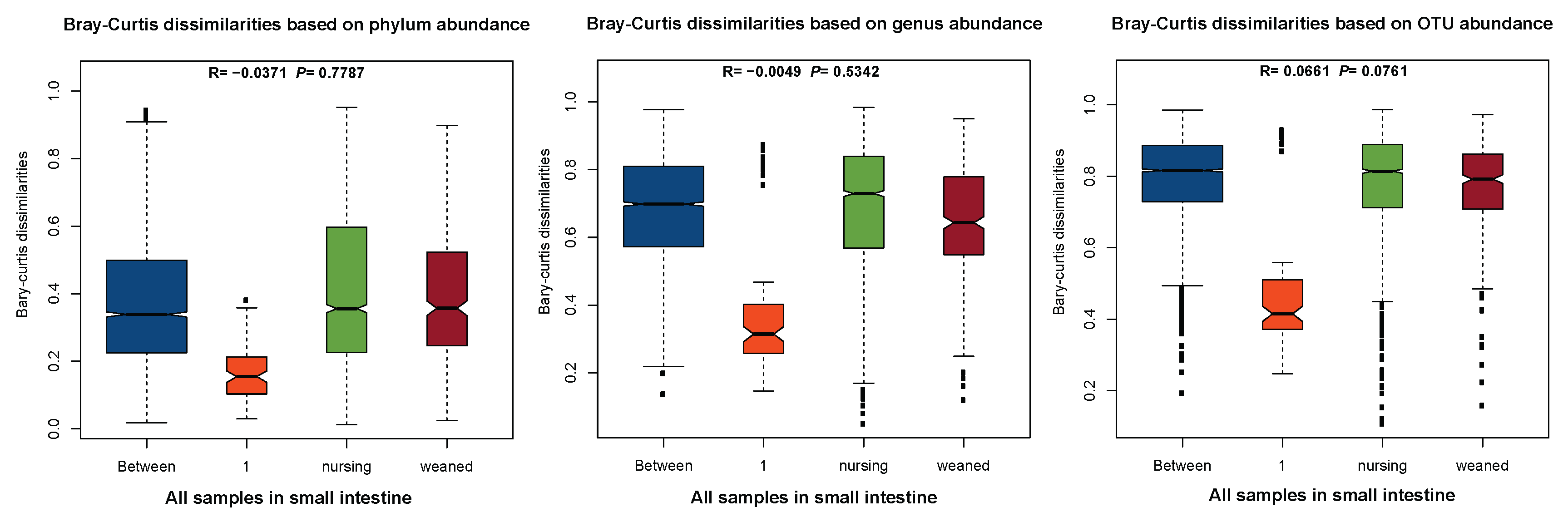
**

**Figure S10.** Bray-Curtis dissimilarities was calculated for the small intestine at three time points, namely day 1, nursing period and weaning period based on the phylum abundance (a), genus abundance (b) and OTU abundance (c).


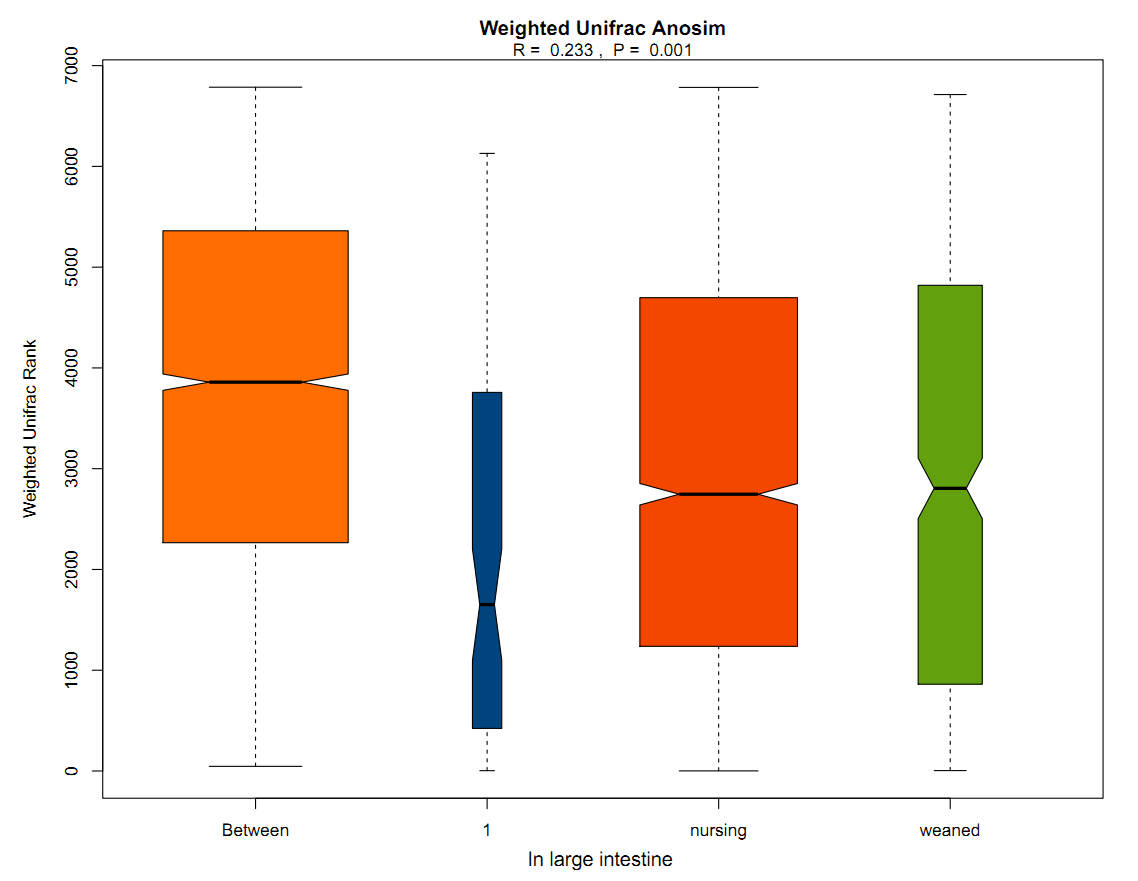


**Figure S11.** The Weighted Unifrac distances revealed that there was significant difference among day 1, nursing period and weaning period in large intestine (R = 0.233, *p* = 0.001).


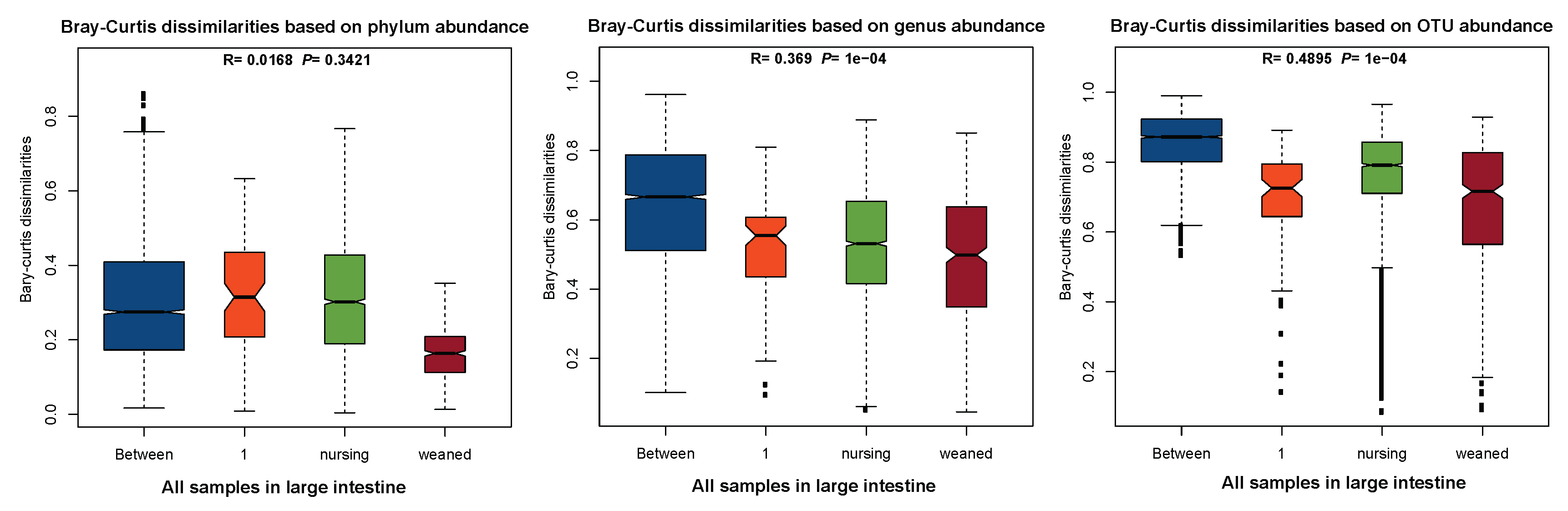


**Figure S12.** Bray-Curtis dissimilarities was calculated for the large intestine at three time points, namely day 1, nursing period and weaning period based on the phylum abundance (a), genus abundance (b) and OTU abundance (c).


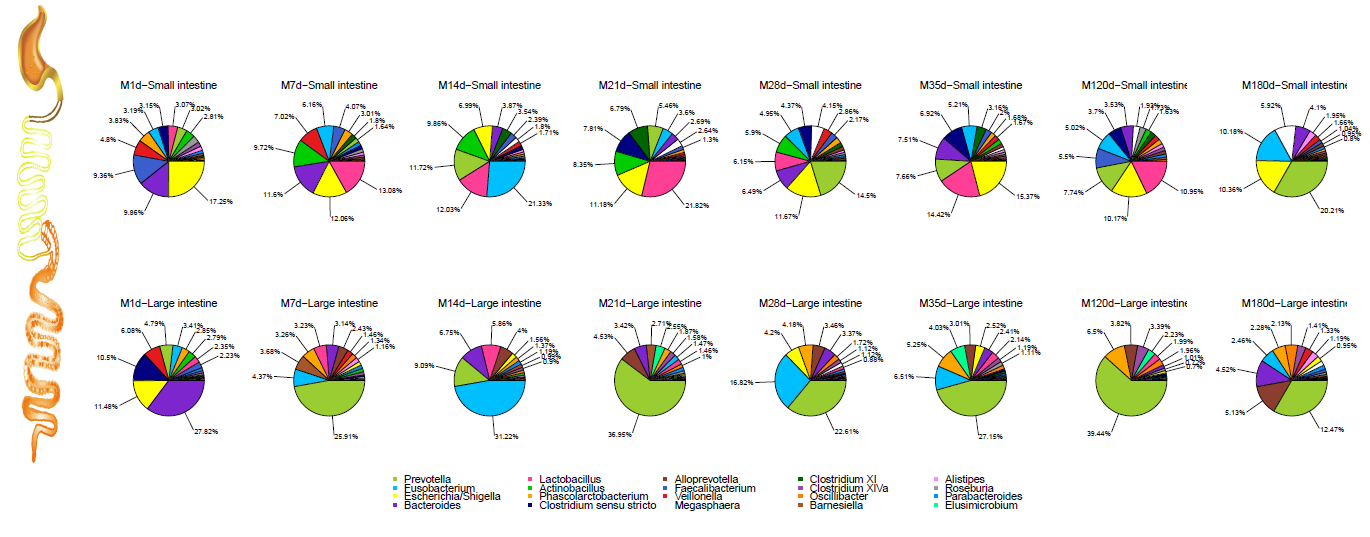
**Figure S13.** Gut microbiota composition at the genus level in the small intestine and large intestine in different growth stages. The first row represents the results of the small intestine, the second row represents the results of the large intestine. The eight columns represent eight time points, namely postnatal day 1, day 7, day 14, day 21, day 28, day 35, day 120 and day 180, from left to right. Each pie chart indicates the composition of the gut microbiome in an intestinal segment at a time point. Different colors represent different bacterial genus.


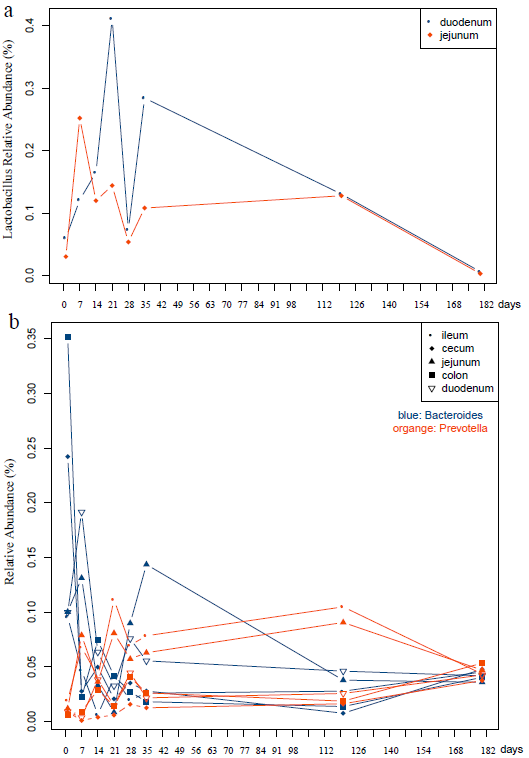


**Figure S14.** The relative abundance of selected phylotypes that showed responses to dietary changes. **a** The dynamics of *Lactobacillus* in duodenum (blue) and jejunum (red). **b** The dynamics of *Bacteroides* (blue) and *Prevotella* (red) different intestine segment. Different symbols denote different intestinal segments.


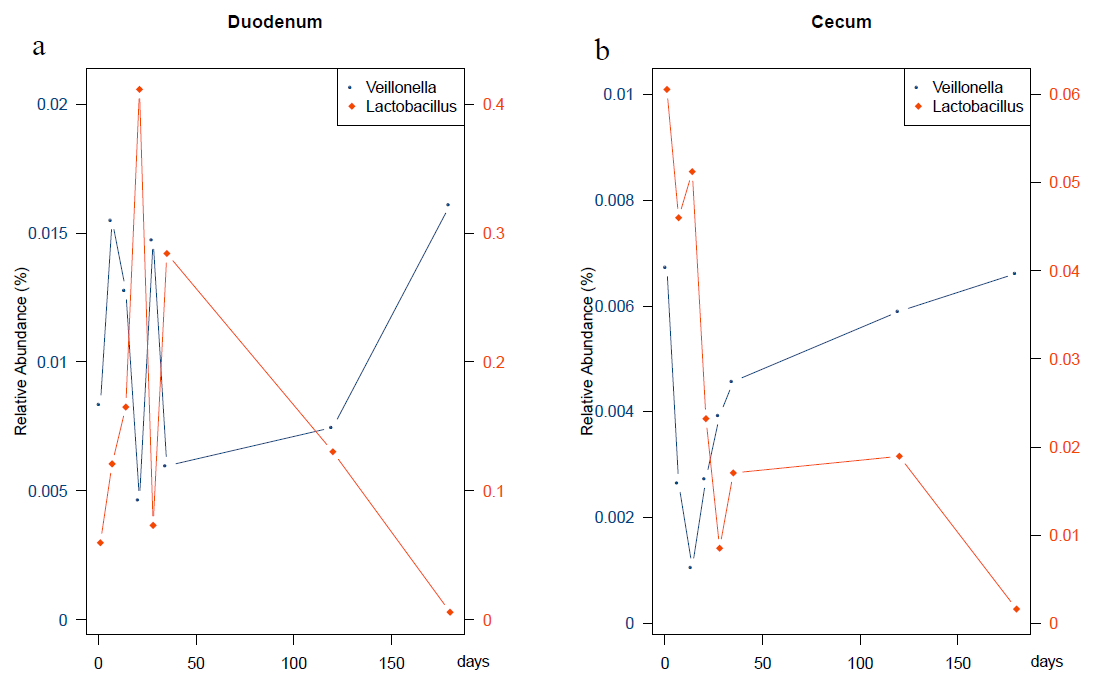


**Figure S15.** The relative abundance curves of two genera in response to dietary changes. **a** The dynamics of *Veillonella* (blue) and *Lactobacillus* (red) in the duodenum. **b** The dynamics of *Veillonella* (blue) and *Lactobacillus* (red) in the cecum.


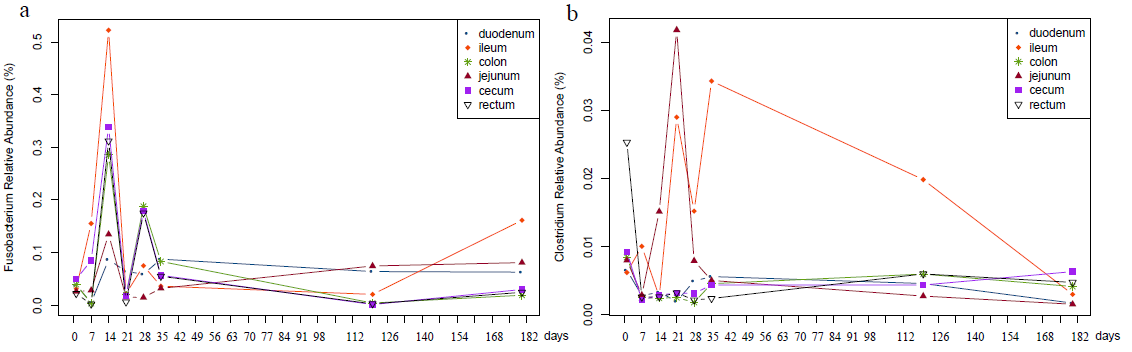


**Figure S16.** The relative abundance curves of two opportunistic pathogens. **a** The dynamics of *Fusobacterium* in different intestinal segments. **b** The dynamics of *Clostridium* in different intestinal segments. Different colors represent individual intestinal segments.


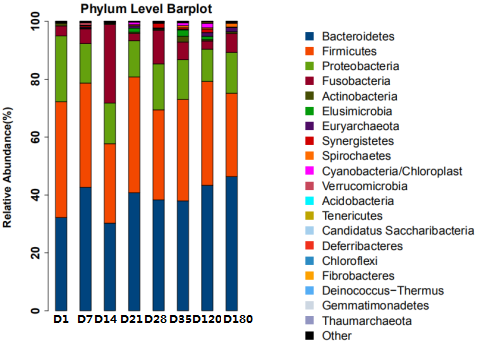


**Figure S17.** Community composition at the phylum level of the gut microbiota in different growth stages of Meishan pigs.
